# N-glycoproteomics of brain synapses and synaptic vesicles

**DOI:** 10.1016/j.celrep.2023.112368

**Published:** 2023-04-09

**Authors:** Mazdak M. Bradberry, Trenton M. Peters-Clarke, Evgenia Shishkova, Edwin R. Chapman, Joshua J. Coon

**Affiliations:** 1 Department of Chemistry, University of Wisconsin-Madison, Madison, WI 53706, USA; 2 Department of Biomolecular Chemistry, University of Wisconsin-Madison, Madison, WI 53706, USA; 3 National Center for Quantitative Biology of Complex Systems, Madison, WI 53706, USA; 4 Howard Hughes Medical Institute and Department of Neuroscience, University of Wisconsin School of Medicine and Public Health, Madison, WI 53705, USA; 5 Department of Psychiatry, Columbia University, New York, NY 10032, USA; 6 Morgridge Institute for Research, Madison, WI 53715, USA; 7 Lead contact

## Abstract

At mammalian neuronal synapses, synaptic vesicle (SV) glycoproteins are essential for robust neurotransmission. Asparagine (***N***)-linked glycosylation is required for delivery of the major SV glycoproteins synaptophysin and SV2A to SVs. Despite this key role for ***N*-g**lycosylation, the molecular compositions of SV ***N***-glycans are largely unknown. In this study, we combined organelle isolation techniques and high-resolution mass spectrometry to characterize ***N***-glycosylation at synapses and SVs from mouse brain. Detecting over 2,500 unique glycopeptides, we found that SVs harbor a distinct population of oligomannose and highly fucosylated ***N***-glycans. Using complementary fluorescence methods, we identify at least one highly fucosylated ***N***-glycan enriched in SVs compared with synaptosomes. High fucosylation was characteristic of SV proteins, plasma membrane proteins, and cell adhesion molecules with key roles in synaptic function and development. Our results define the ***N***-glycoproteome of a specialized neuronal organelle and inform timely questions in the glycobiology of synaptic pruning and neuroinflammation.

## Graphical abstract

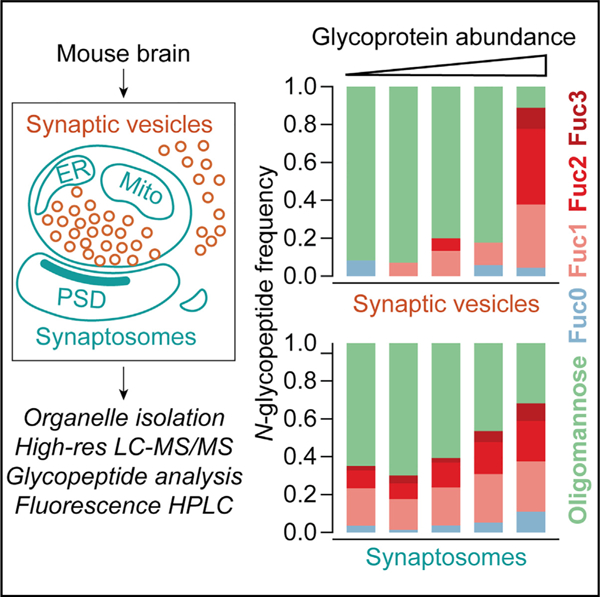

## In brief

Bradberry et al. combine organelle immunopurification and orthogonal chemical analysis methods to characterize protein ***N***-glycosylation at the murine brain synapse. Their results define a glycobiological signature of brain synaptic vesicles and demonstrate that synaptic vesicle and plasma membrane proteins bear highly fucosylated ***N***-glycans.

## INTRODUCTION

Synaptic vesicles (SVs) are small recycling organelles that store and release neurotransmitters at nerve terminals. Post-translational modifications of SV proteins may play important roles in SV formation and function.^1–4^ Among these modifications, glycosylation of luminal asparagine residues (*N*-linked glycosylation) is integral to membrane protein folding and trafficking.^5,6^ Several abundant SV proteins with established roles in neurotransmitter release are *N*-glycoproteins, including the Ca^2+^ sensor synaptotagmin-1,^7–9^ the endocytosis-related tetraspanin synaptophysin,^10,11^ and the SV2A/B/C family of transporter-like glycoproteins.^12–15^ SV2A and synaptophysin both require intact glycosylation sites for normal trafficking,^16^ and glycosylation of SV2C is essential for the uptake of botulinum neurotoxin A.^17^

Compared with plasma and other tissues, the brain *N*-glycome is rich in oligomannose and bisected N-acetylglucosamine (GlcNAc)-rich structures .^18–23^ The brain *N*-glycome is dynamic over the lifespan,^20,24^ and blocking neuronal *N*-glycan maturation results in a striking neurodegenerative phenotype.^25^ Moreover, evidence suggests that certain glycoepitopes may be important for brain development and function. For example, loss of the enzyme fucosyltransferase-9 (Fut9),^26^ which generates Lewis^X/SSEA-1/CD15^ glycoepitopes^27,28^ in mouse brain by addition of antennary fucose in α(1–3) linkages to complex *N*-glycans,^29,30^ is associated with impaired neurite outgrowth *in vitro*^31^ and behavioral abnormalities in the mouse.^29^ Strikingly, the corresponding gene *FUT9* has been linked to schizophrenia in human genome-wide association studies, along with several other glycosylation-related genes.^32–34^ Aberrant glycosylation has long been detected in the brains of patients with schizophrenia, though largely through the use of indirect methods such as immunoblotting and immunostaining.^35^ These findings suggest that glycobiological processes may contribute to the development of schizophrenia, but specific pathophysiologic mechanisms remain poorly understood in this context.

Recent advances in mass spectrometry-based glycoproteomics methods have enabled the detection of hundreds to thousands of unique glycopeptides obtained from brain tissue.^36–40^ While these studies have improved our understanding of the heterogeneous distribution of *N*-glycosylation in the brain,^39^ their broad scope has limited opportunities for insight into roles for specific glycans in specific biological processes. Examining protein glycosylation in particular cell types or organelles, by contrast, offers an opportunity to link glycosylation with function. In particular, the abundance of glycoproteins in SVs^41–43^ makes these organelles attractive for the study of glycans in membrane protein trafficking. Moreover, nerve terminals represent a brain tissue component vulnerable to injury and degeneration, especially in the context of neuroinflammation,^44–46^ schizophrenia,^47^ and age-related cognitive decline.^48–50^ Changes in glycoprotein trafficking at the synapse may thus influence a broad range of clinically important processes. We also note a large body of evidence suggesting specialized extracellular matrix glycans at nerve terminals.^51^ A meaningful understanding of glycobiology at the synapse, however, has been limited by the lack of a deeply characterized presynaptic *N*-glycoproteome.

In this study, we establish a molecular foundation for synaptic glycobiology by defining the *N*-linked glycoproteome of SVs and their milieu. Using organelle immunoisolation techniques^41^ and complementary analytical methods, we demonstrate that SVs are enriched for specific *N*-glycans bearing antennary fucosylation. Highly fucosylated glycans were found primarily on SV proteins, cell adhesion molecules with known synaptic functions, and other proteins with key roles at the plasma membrane, including transporters and neurotransmitter receptors. Our results define the glycoproteome of a neuronal organelle, suggest a common glycosylation pathway shared by plasma membrane and SV proteins, and provide protein-level evidence to inform hypotheses linking protein glycosylation and synaptic biology.

## RESULTS

### Proteomic characterization of SVs and their milieu

**Figure 1.**
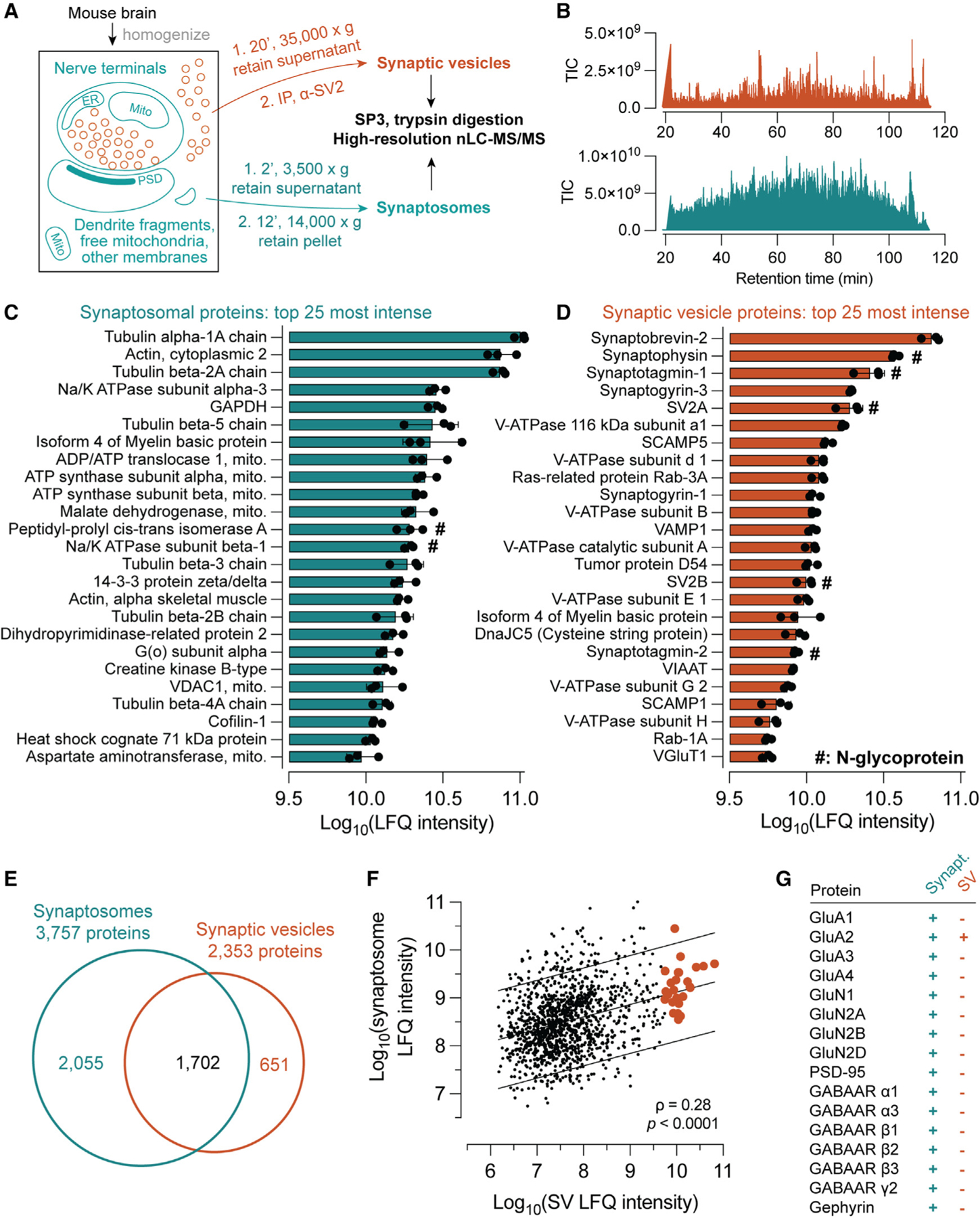
Proteomic characterization of synaptic vesicles and synaptosomes (A) Purification scheme. Synaptic vesicles (SVs) were isolated using magnetic beads conjugated in-house to a monoclonal α-SV2 antibody, while synaptosomes were isolated by differential centrifugation. Both sample types underwent similar processing methods and identical LC-MS/MS analysis procedures. (B) Example MS^1^ total ion current (TIC) chromatograms for SV and synaptosome samples. (C) The top 25 most intense proteins by label-free quantification (LFQ) analysis in synaptosome samples. Cytoskeletal and mitochondrial proteins predominated, consistent with contributions from multiple subcellular components. LFQ intensities were calculated using the top 3 peptides for each protein. Data are represented as mean ± SD from three biological replicates. (D) In contrast, the top 25 most intense proteins in the SV preparation are largely well established to reside on SVs, with only one putative contaminant (myelin basic protein) observed among them. (E) Venn diagram of proteins detected in synaptosome and SV samples, demonstrating the expected high degree of overlap. (F) LFQ intensities of synaptosomal and SV proteins are positively correlated (Spearman’s rho = 0.28, p < 0.0001), consistent with a contribution of SVs to the synaptosomal proteome. Orange points indicate proteins listed in (D), and lines indicate best fit with 90% prediction bands. (G) Ionotropic glutamate and GABA receptor subunits and post-synaptic scaffolding proteins were detected in synaptosomes but were largely absent from SV samples, demonstrating the purity of SVs and the inclusion of post-synaptic material in the synaptosome sample. See also Table S1, which contains all protein-level identification and quantification data used to generate this figure.

Proteomic, glycoproteomic, and *N*-linked glycomic studies were conducted using synaptic material from whole mouse brain prepared by two approaches. Synaptosomes, which represent a “classical” crude preparation containing mostly preand post-synaptic elements, were prepared by differential centrifugation (Figure 1A).^42^ Along with synaptosomes, a highly pure population of SVs was isolated by immunoprecipitation (IP) with magnetic beads conjugated in-house to a monoclonal antibody (mAb) against SV2,^13^ according to recently described procedures (Figure 1A).^41,52^ These preparations were subjected to proteomic analysis by trypsin digestion and nano-flow liquid chromatography-tandem mass spectrometry (nLC-MS/MS) using an Orbitrap Eclipse mass spectrometer^41^ (Figure 1B). Protein abundances were estimated by label-free quantification (LFQ) analysis. The synaptosome preparation was rich in cytosolic and mitochondrial proteins (Figure 1C; Table S1), consistent with a low degree of specific enrichment for any particular cellular compartment. In contrast, SV2-IP yielded SVs of exceptionally high purity, as evinced by the nearly exclusive presence of well-established SV proteins^41,42^ among the top 25 most intense in this preparation (Figure 1D; Table S1). More than 2,300 proteins were detected in this SV preparation, among which over 1,700 were also identified in the synaptosome fraction (Figure 1E). This represents the largest number of proteins detected in a highly pure SV preparation to date.^41,53^ Comparison of SV2-IP SVs with a previously reported Syt1-IP preparation^41^ (Figure S1) demonstrates robust correlation of protein LFQ intensities obtained using each antibody, confirming the similarity of Syt1-IP and SV2-IP preparations. Both SV2-IP and Syt1-IP yield “general” samples of brain SVs containing primarily glutamatergic and GABAergic, but also monoaminergic and cholinergic, vesicle types (Figure S1). LFQ intensity scores of synaptosome and SV proteins were positively correlated (Figure 1F), consistent with an expected contribution from SVs to the synaptosomal proteome.

SVs were largely free of contamination from post-synaptic membranes (Figure 1G). Type-A gamma-amino butyric acid (GABA_A_), N-methyl-D-aspartate (NMDA), and α-amino-3-hydroxy-5-methyl-4-isoxazolepropionic acid (AMPA) receptor subunits were present in synaptosomes but were largely undetectable in SV samples (Figure 1G). SV samples likewise did not contain the post-synaptic scaffolding proteins PSD-95 or gephyrin, both of which were present in synaptosome samples (Figure 1G). These results establish that our synaptosome preparation contains not just mature SVs but also a more general sample of brain proteins that represents the synaptic milieu.

**Figure 2.**
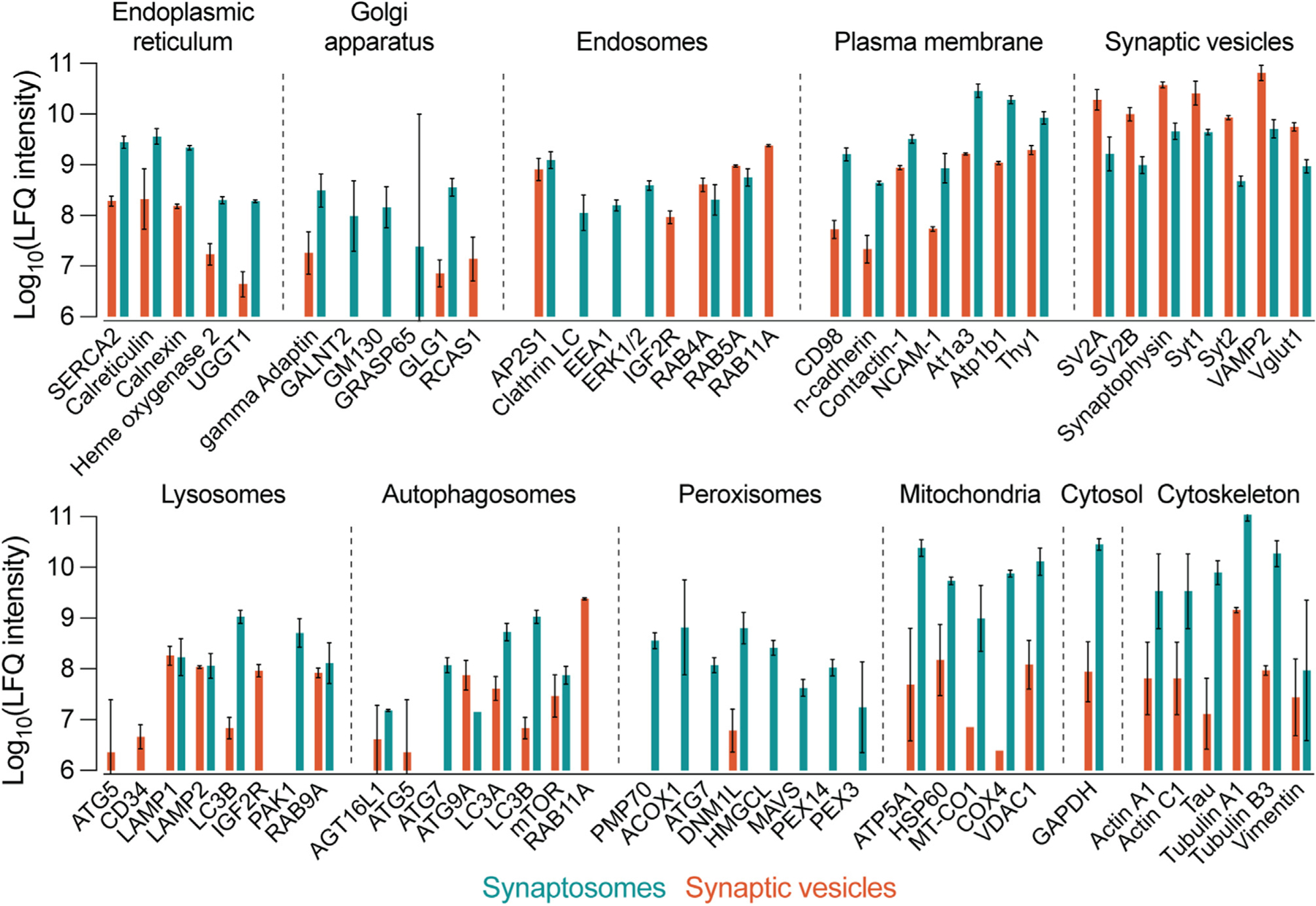
Proteomic survey of organelle markers in SVs and synaptosomes LFQ results were sorted and grouped using established organelle marker proteins. Bars represent the mean LFQ intensity values for three replicates in SV or synaptosome samples. Marker proteins were included if they were quantified in at least two of three replicates for SVs or synaptosomes. Compared with synaptosomes, SVs were more abundant in SV markers and less abundant in most other markers by approximately one to two orders of magnitude in each direction. Exceptions to this included some lysosomal and endosomal markers such as LAMP and Rab proteins. Synaptosomes were markedly more abundant in cytosol, cytoskeleton, endoplasmic reticulum, plasma membrane, and mitochondria markers compared with SVs. SVs were also largely devoid of peroxisome markers. Data are presented as mean ± 95% confidence interval (CI) from two to three biological replicates. See also Table S1, which contains all protein-level identification and quantification data used to generate this figure.

SV and synaptosome samples were further characterized by sorting for proteins commonly used to mark a variety of organelles (Figure 2). In agreement with Figures 1C and 1D, SV markers predominated in SV samples, while most markers for other organelles were substantially more abundant in synaptosomes (Figure 2). This difference was particularly apparent for endoplasmic reticulum (ER), Golgi, plasma membrane, peroxisome, and mitochondria markers. By contrast, endosomal and lysosomal markers were more varied in their distribution, with Rab and LAMP proteins more evenly distributed between SVs and synaptosomes. These results accord with prior characterizations of the SV proteome^41,42,53^ and suggest that some protein machinery is shared between the SV and endolysosomal membrane systems. Figure 2 demonstrates that synaptosomes represent a broad survey of the neuronal secretory pathway in addition to pre- and post-synaptic membranes (Figure 1).

### Glycoproteomics at the synapse

**Figure 3.**
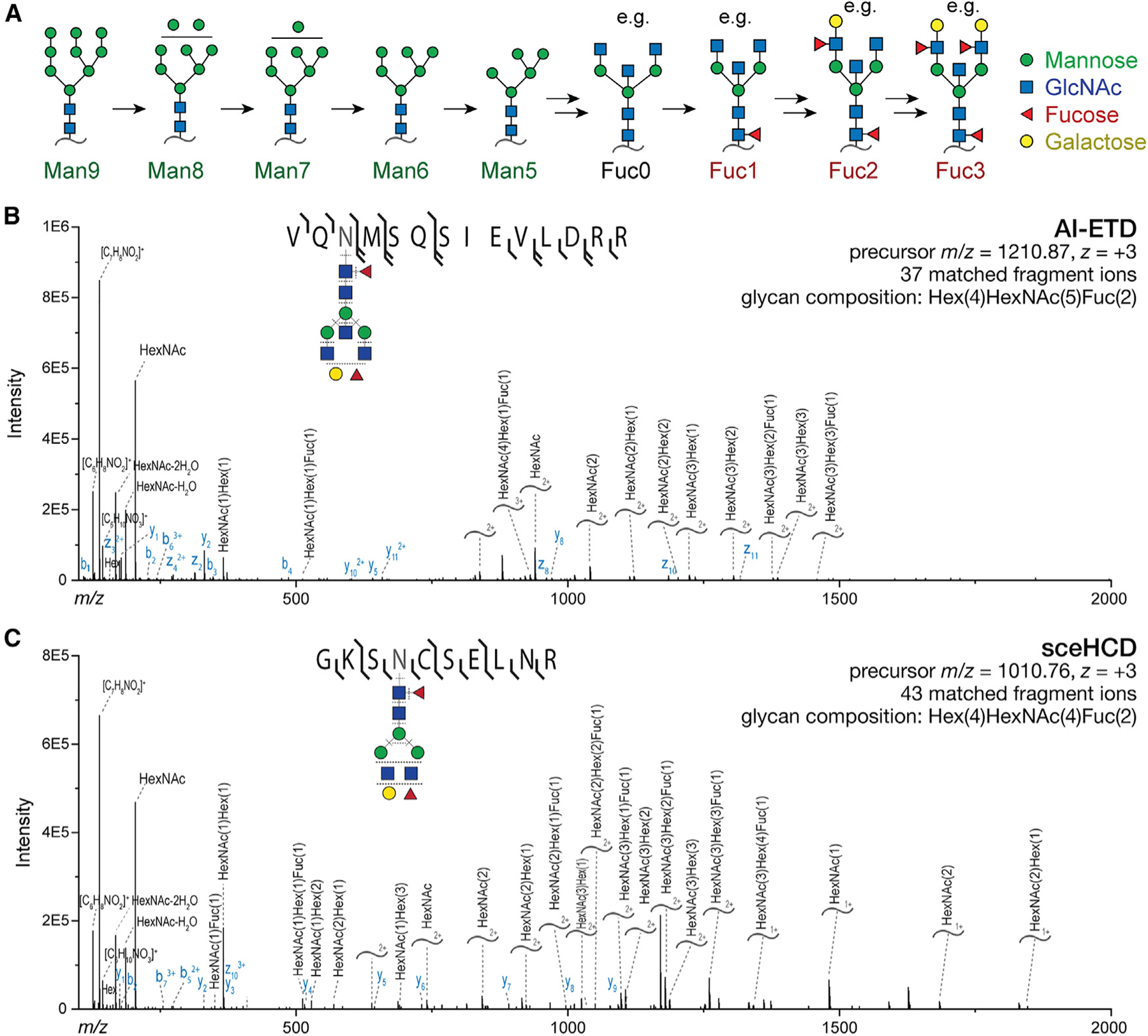
Characterization of protein *N*-glycosylation by MS/MS (A) Illustration of the *N*-glycan maturation sequence for the predominant brain *N*-glycans. Many potential glycans contain 0–3 fucoses, with some examples shown here. (B) Illustrative annotated AI-ETD spectrum with glycopeptide precursor shown. Curved lines annotated with glycan compositions denote *Y* ions, which contain the intact peptide and a fragmented glycan. Both *b/y* and *c/z* ions are detected owing to hybrid vibrational and electron transfer modes of fragmentation. (C) As in (B) but with sceHCD fragmentation of the depicted glycopeptide, which yields *b*/*y* and *Y* ions.

With the contents of synaptosomes and SVs established, we turned our attention to the *N*-glycosylation of proteins in these two preparations. Several studies have established that brain N-glycans comprise mostly mannosylated glycans and fucosylated complex glycans bearing bisecting GlcNAc^19–22,54^ (Figure 3A). Importantly, most mannose sugars are usually removed from the glycan before further addition of GlcNAc,^6,22^ and fucose is typically first added in an α(1,6) linkage to the *N*-linked core GlcNAc before the addition of antennary fucose in α(1,3) or α(1,4) linkages (Figure 3A).^22^ In this study, protein *N*-glycosylation was characterized by fragmentation of glycopeptides in an Oribtrap mass spectrometer (Figures 3B and 3C), which enables site-specific characterization of *N*-glycosylation on tryptic glycopeptides. Fragmentation was achieved with either activated-ion electron transfer dissociation (AI-ETD)^39,40,55,56^ (Figure 3B) or stepped collision energy higher-energy collisional dissociation (sceHCD)^57^ (Figure 3C).

**Figure 4.**
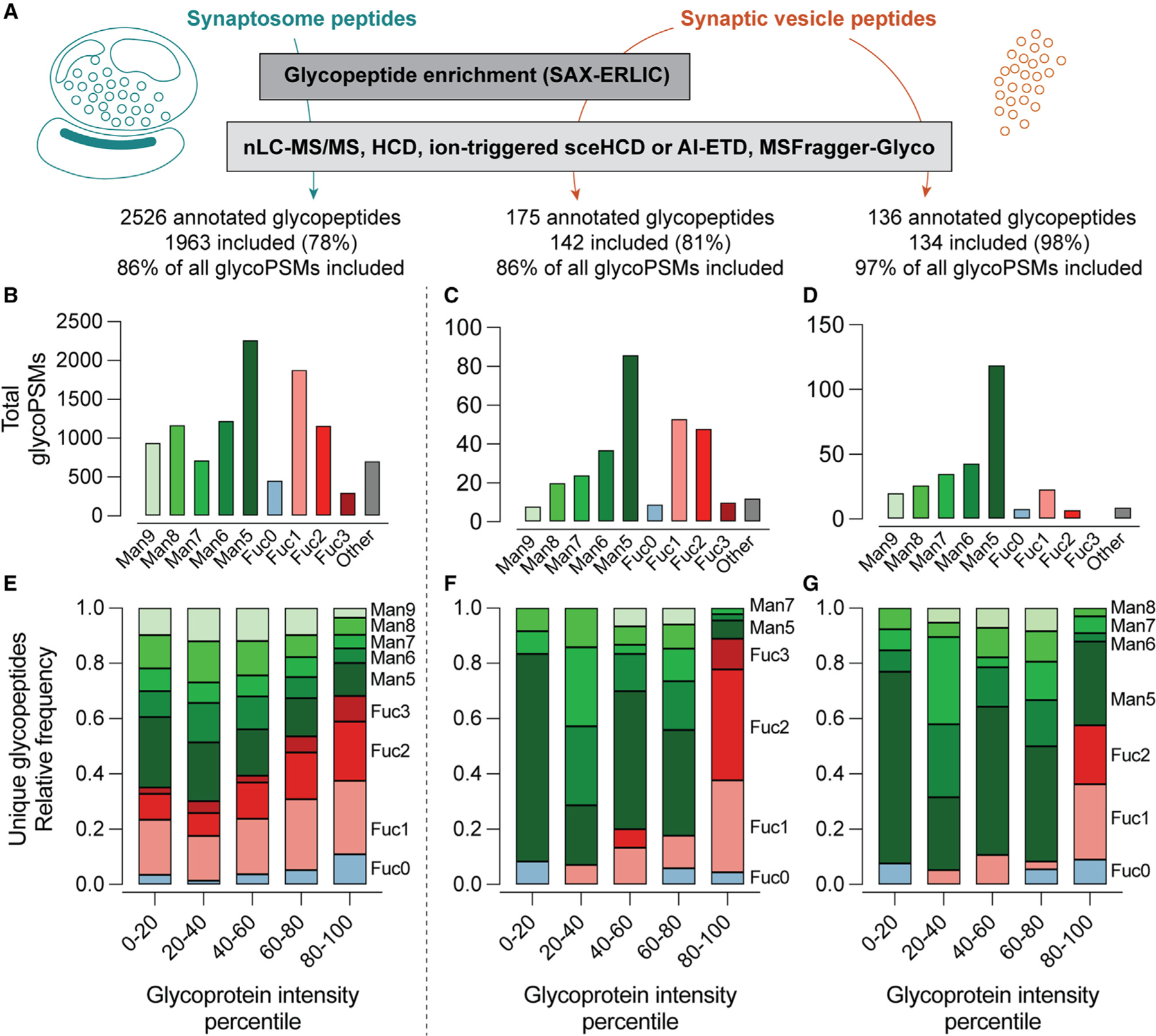
Glycoproteomics at the synapse (A) Analysis scheme. Tryptic peptides, with or without glycan enrichment by SAX-ERLIC, were analyzed using a fragmentation strategy employing sceHCD or AI-ETD. Glycans were included if they could confidently be assigned as oligomannose, complex, or hybrid N-glycans, and sialylated glycans were excluded. (B–D) Distribution of all annotated glycan peptide spectral matches (glycoPSMs) meeting inclusion criteria. Antibody-derived glycopeptides were omitted from analysis. No Fuc3 peptides were identified in the non-enriched SV samples. (E–G) Distribution of unique annotated glycopeptides according to intensity quintile of the corresponding glycoproteins determined by standard proteomics methods (see Figure 1). SV samples demonstrate a marked increase in Fuc2 and Fuc3 sugars in the highest quintiles, while the distribution of glycopeptides in synaptosome samples was biased to a lesser degree (p < 0.0001, SV versus synaptosome samples, Mann-Whitney test). See also Table S2, containing MSFragger search output; Table S3, containing all annotated glycoPSMs used to generate this figure; Table S4, which was used to annotate glycoPSMs according to Man or Fuc content; Table S5, which contains protein LFQ intensity values and quintile assignments used to generate (E)–(G); and Figures S2 and S3, which provide additional compositional data for synaptosome and SV glycoPSMs.

To obtain *N*-glycoproteomes for SVs and synaptosomes, we performed nLC-MS/MS experiments employing glycopeptide enrichment and glycan-focused fragmentation approaches. Synaptosome samples were characterized by both AI-ETD- and sceHCD-based methods after glycopeptide enrichment using strong anion exchange-electrostatic repulsion chromatography (SAX-ERLIC) prior to nLC-MS.^58^ SV samples were characterized using sceHCD both with and without glycopeptide enrichment (Figure 4A) given the lower complexity and abundance of these samples (Figure 1). Importantly, SAX-ERLIC is less specific for particular *N*-glycans than lectin affinity enrichment methods,^58^ reducing the potential for bias toward certain glyocopeptides among enriched samples. In combination with the MSFragger/FragPipe analysis pipeline,^38,59^ these data enabled a rich glycoproteomic survey of synaptosomes and SVs purified from whole mouse brain (Figures 4A–4D). Glycan peptide spectral matches (GlycoPSMs) were obtained by analysis with MSFragger in glyco mode^38,60^ (Table S2) and filtered by excluding those glycoPSMs with a calculated false discovery rate cutoff (Q value) of >0.025. A total of over 2,500 unique glycopeptides from over 550 glycoproteins were identified by this method (Figure 4A; Table S3), with the majority observed in the substantially more complex synaptosome samples (Figure 4A). The annotated glycopeptides were analyzed based on their degree of mannosylation or fucosylation (Figure 3A) with guidance from recently published work describing the composition of the brain *N*- and *O-*glycomes^19,20,22^ (Figures S2 and S3; Table S4). GlycoPSMs containing sialic acid or unusually large numbers of sugars were excluded from analysis, as we inferred that these glycoPSMs could correspond to peptides bearing both *N*- and *O*-glycosylation on the same tryptic peptide given the low prevalence of sialylated *N*-linked glycans, and the high prevalence of sialylated *O*-linked glycans, in the brain.^22^ Despite these limitations, over 85% of glycoPSMs were included (Figures S2 and S3). Distributions of glycoPSMs, corresponding to major *N*-glycan types shown in Figure 3A, are shown in Figures 4B–4D. In agreement with the work of Williams et al.,^22^ we observed a predominance of oligomannose species along with fucosylated complex species (Figures 4B–4D), with a minor contribution from glycans bearing two or more fucoses. Proteins bearing three fucoses (Fuc3) were not observed in SV samples without glycopeptide enrichment (Figure 4D), likely due in part to poorer positive-mode ionization efficiency of peptides bearing larger glycans.

While informative, the distribution of glycoPSMs may not reflect the actual composition of *N*-glycans in SVs or synaptosomes, as these data do not account for differences in abundance among glycoproteins giving rise to these glycopeptides. We thus analyzed these data further by considering the LFQ intensity scores obtained in our standard proteomics experiments (Figure 1) for each identified glycoprotein (Figures 4E–4G; Table S5). Glycoproteins were grouped into quintiles based on their intensity values (Figure 1), and the distribution of glycoPSMs was analyzed for each quintile in each sample type (Figures 4E–4G). This analysis revealed a striking bias toward fucosylation in the most abundant SV glycoproteins (Figures 4F and 4G), which was less pronounced in the synaptosome samples (Figure 4E). This trend was particularly evident for glycans containing two or three fucoses (i.e., highly fucosylated glycans) (Figures 4E–4G). In glycan-enriched SV samples, nearly all the unique glycoPSMs for the most abundant SV glycoproteins contained one or more fucoses (Figure 4F). A bias toward fucosylated glycopeptides in the most abundant SV proteins was observed whether or not glycopeptide enrichment was used (Figures 4F and 4G), demonstrating that this observation is not an artifact of the enrichment procedure. However, this bias toward high fucosylation in abundant SV proteins was best observed using glycopeptide enrichment (Figure 4F). Together, these results suggest that abundant SV proteins are rich in highly fucosylated *N*-glycans.

### Characterization of SV and synaptosome glycans by HILIC-HPLC

**Figure 5.**
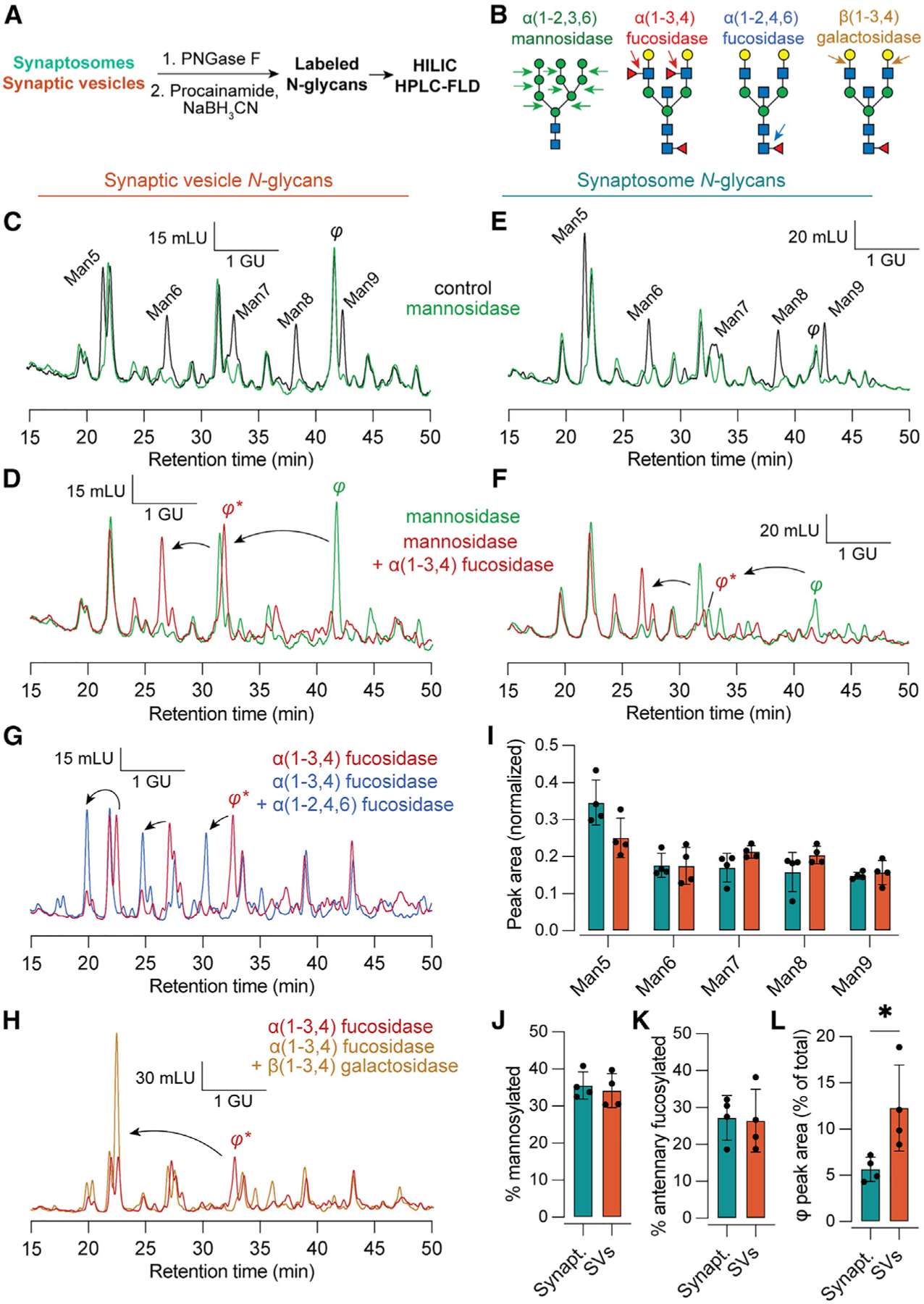
Fluorescence HPLC of released *N*-glycans defines a Fuc3 species enriched on SVs (A) Experimental scheme. (B) Illustration of cleavage sites for exoglycosidases used for this figure. α(1–2,3,6) mannosidase cleaves mannose residues from mannosylated *N*-glycans. α(1–3,4) fucosidase removes only antennary fucose but not the core fucose, which is sensitive to α(1–2,4,6) fucosidase. β(1–3,4) galactosidase removes antennary galactose residues. (C) Representative chromatograms of procainamide-labeled SV *N*-glycans under control conditions and treated with mannosidase. The identity of the mannose series was established using an authentic Man5 standard. Mannosidase-sensitive peaks appear in black. A mannosidase-insensitive peak eluting between Man8 and Man9 is labeled *φ*. GU, glucose units, determined via procainamide-labeled glucose homopolymer standard; mLU, milli-luminance units. (D) The addition of α(1–3,4) fucosidase causes *φ* to migrate ~1.9 GU, indicating the presence of two antennary fucoses. (E and F) As in (C) and (D) but for synaptosome *N*-glycans. While a peak corresponding to *φ* is observed in both sample types, this species is relatively more abundant in SVs. (G) After treatment with α(1–3,4) fucosidase, *φ* remains sensitive to α(1–2,4,6) fucosidase, consistent with α(1–6) core fucosylation and a total of 3 fucoses. (H) *φ* was also sensitive to β(1–3,4) galactosidase, which caused an additional ~2 GU shift. (I–K) The distribution of peak areas among mannosylated glycans, as well as the total contribution from mannosylated and antennary fucosylated glycans, was similar in synaptosomes and SVs. (L) The peak corresponding to *φ* made a significantly larger contribution to SV versus synaptosome glycans (p = 0.02, Mann-Whitney test), demonstrating enrichment of this Fuc3 glycan in SVs. Data are represented as mean ± SD from four biological replicates. See also Figure S5, which contains additional raw HPLC data used to characterize peaks corresponding to Man5–9 and *φ*.

While the above results provide evidence that abundant SV glycoproteins are biased toward fucosylation, additional caveats exist to the interpretation of bottom-up glycoproteomics data. For example, unexpected biases in the positive-mode ionization efficiency of certain glycopeptides could cause skewed results.^61^ We thus employed an orthogonal, ionization-independent *N*-glycan analysis method involving fluorescent detection of isolated glycans from SV and synaptosome samples (Figure 5A). *N*-glycans were specifically and quantitatively cleaved from proteins using PNGase F (Figure S4), followed by labeling with the fluorescent drug procainamide via reductive amination (Figure 5A). Labeled *N*-glycans were analyzed by amide hydrophilic interaction chromatography with fluorescence detection (HILIC-HPLC-FLD) (Figure 5). Digestion with exoglycosidases specific for mannose, antennary fucose, or galactose (Figure 5B) enabled a semi-quantitative determination of the contributions of various glycans to the SV and synaptosomal *N*-glycomes (Figures 5C–5L). The identities of the mannosylated glycans were determined using a Man5 standard and glucose homopolymer ladder, defining a clear sequence of glycans bearing 5–9 mannose residues (Figure S5). Among mannosylated glycans, we observed a trend toward predominance of Man5 in both synaptosomes and SVs (Figures 5C, 5E, and 5I), but we did not observe statistically significant differences between the two sample types either in the distribution of mannosylated glycans (Figure 5I) or in the total contribution from mannosylated glycans (Figure 5J).

In accordance with our LC-MS glycoproteomics data (Figure 4), both SV and synaptosomal samples contained a major contribution from antennary fucosylated glycans (Figures 5D, 5F, and 5K), equal to ~25% of all *N*-linked glycans. Strikingly, a late-eluting antennary fucosylated species bearing two antennary fucose residues, denoted *φ*, was significantly enriched in the SV samples (Figures 5C–5F and 5L). This peak was susceptible to further cleavage when incubated with α(1–2,4,6) fucosidase (Figure 5G), demonstrating that this species is also core fucosylated and thus contains 3 total fucoses. This peak was also susceptible to cleavage with β(1–3,4) galactosidase, which caused another ~2 glucose unit shift in the elution time of this peak (Figure 5H). The resulting peak was sensitive to cleavage with β-GlcNAcase (Figure S5). Examination of our glycoproteomics data (Figures 4 and S3; Table S3) demonstrated a predominant Fuc3 species, HexNAc(5)Hex(5)Fuc(3), on the abundant SV glycoproteins SV2B and synaptophysin in glycopeptide-enriched SV samples. While this glycan composition could account for *φ*, further work is needed to ascertain the precise molecular identity of this SV-enriched glycan. We did not observe sensitivity to α(1–2) fucosidase among synaptosome or SV glycans (Figure S5), consistent with low expression of the corresponding fucosyltransferase enzymes Fut1 and Fut2 in mouse brain.^22^ Finally, we ensured that our findings were not due to elution of α-SV2 mAb from the magnetic beads, as these SV2 mAb glycans eluted at different times and were not sensitive to α(1–3,4) fucosidase or α(1–2,3,6) mannosidase (Figure S5). These results confirm that both SVs and synaptosomes are rich in antennary-fucosylated glycans, with specific enrichment of at least one Fuc3 species in SVs.

### Deep characterization of the SV glycoproteome

**Figure 6.**
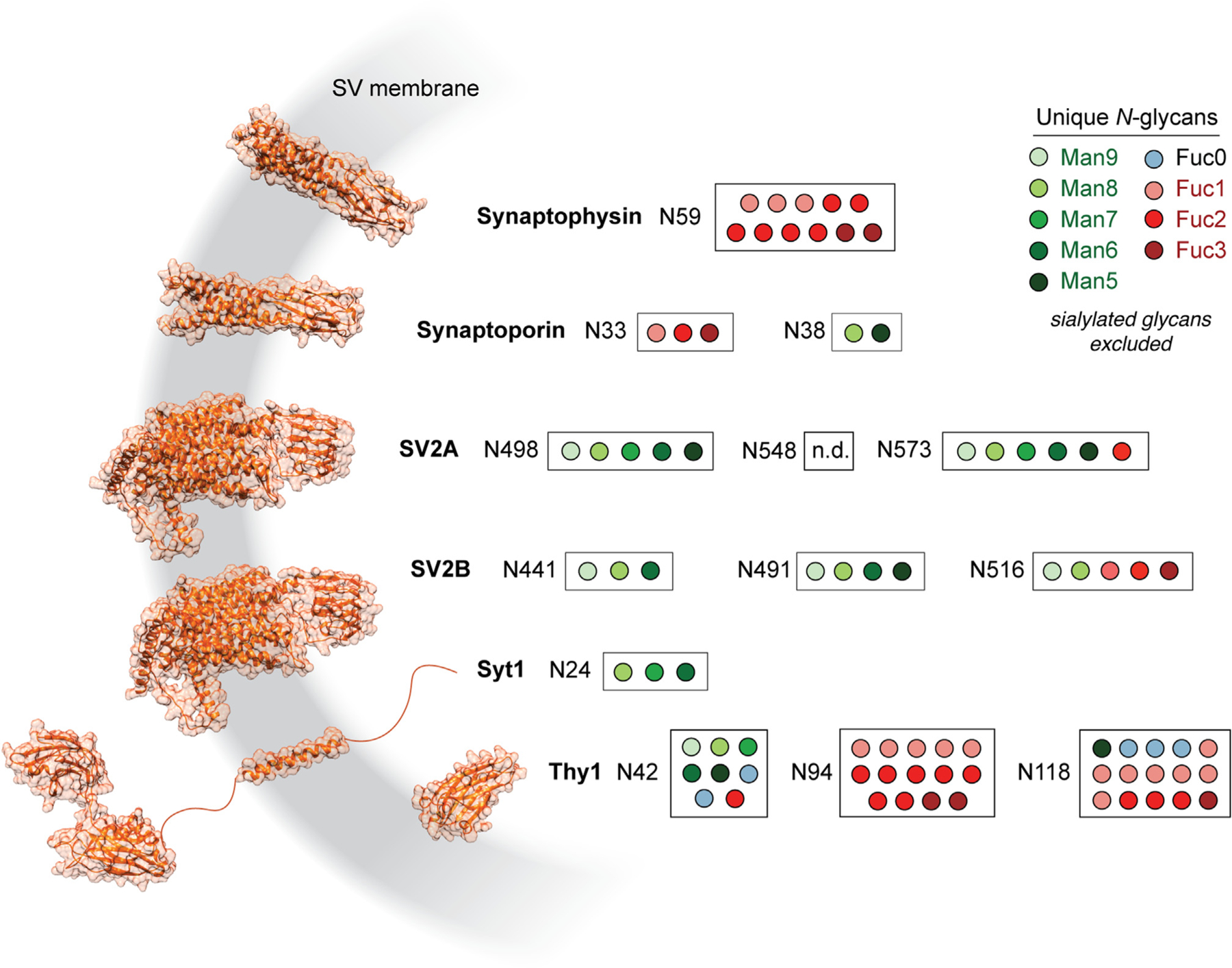
Deep glycoproteomics of the SV reveals site-specific biases toward mannosylation or fucosylation of SV proteins Several of the most abundant SV glycoproteins are depicted with AlphaFold structures^65^ and filled circles corresponding to unique *N*-glycopeptides detected for each *N*-linked glycosylation site. Sites are noted by the amino acid sequence number for each glycosylated asparagine. High fucosylation was observed for all SV glycoproteins shown here except for syt1. SV glycoproteins with multiple glycosylation sites demonstrated a bias toward fucosylation at a single site, while other sites were more likely to bear oligomannose or other non-fucosylated *N*-glycans. No glycoPSMs were observed for peptides containing N548 on SV2A. See also Table S6, which contains the glycoPSM data used to generate this figure, and Figure S6, which addresses prior studies on the glycosylation of SV2.

Previous work has shown that *N*-glycosylation in the brain can be remarkably heterogeneous not only across glycosites for a given protein but also at any given glycosite.^39^ Moreover, while the above results support the enrichment of at least one glycan with antennary fucosylation on SV proteins, SVs still contain a substantial proportion of oligomannose glycans (Figures 4 and 5). A deeper examination of the glycosylation sites on each major SV glycoprotein would yield additional insights into the nature and distribution of protein glycosylation in SVs. We thus immunoprecipitated SV2 and syt1 from detergent-solubilized synaptosomes and analyzed these samples by glycan-targeted LC-MS to characterize *N*-glycosylation more thoroughly for these major SV glycoproteins (Tables S2 and S6). The resulting data were combined with our SV and synaptosome glycoproteomics data (Figure 3; Tables S3 and S6) to define the set of unique glycopeptides present at each glycosite among major SV glycoproteins (Figure 6). In accordance with the work of Riley et al.,^39^ we found that each glycosite could carry any of several unique glycans, and heterogeneity across glycosites was observed for each protein (Figure 6). Strikingly, while high fucosylation was observed on each protein except for syt1, high fucosylation was usually observed on only one site per SV protein (Figure 6). In the case of synaptophysin, which harbors a single glycosylation site, only fucosylated glycans were detected. In SV proteins with multiple glycosylation sites, especially SV2B, non-fucosylated sites harbored oligomannose glycans (Figure 6). We did not observe high molecular weight forms of SV2 previously characterized by immunoblotting as keratan sulfate proteoglycans^62^ (Figure S6). Rather, we found that boiling SV or synaptosome samples caused an apparent increase in the molecular weight of SV2 (Figure S6), suggesting that this early evidence for keratan sulfate on SV2 may represent an experimental artifact. Thy1, which is among the most abundant neuronal plasma membrane glycoproteins and is also found in SVs,^41,63,64^ contained fucosylation at all three glycosites but was likewise biased toward fucosylation at a single site, N94 (Figure 6). By contrast, syt1 was unique in its lack of fucosylation (Figure 6). Indeed, the single *N*-glycosylation site on syt1 was observed to contain at least six mannose residues, even though Man5 was the most common mannosylated glycan observed in each sample (Figures 4 and 5) and in the brain generally.^22^ Together, these results paint a highly resolved picture of the SV glycoproteome, raise new questions about the trafficking itineraries of SV proteins, and suggest important biochemical constraints on protein glycosylation in the Golgi apparatus.

### Fucosylated *N*-glycans are characteristic of SV and plasma membrane proteins

**Figure 7.**
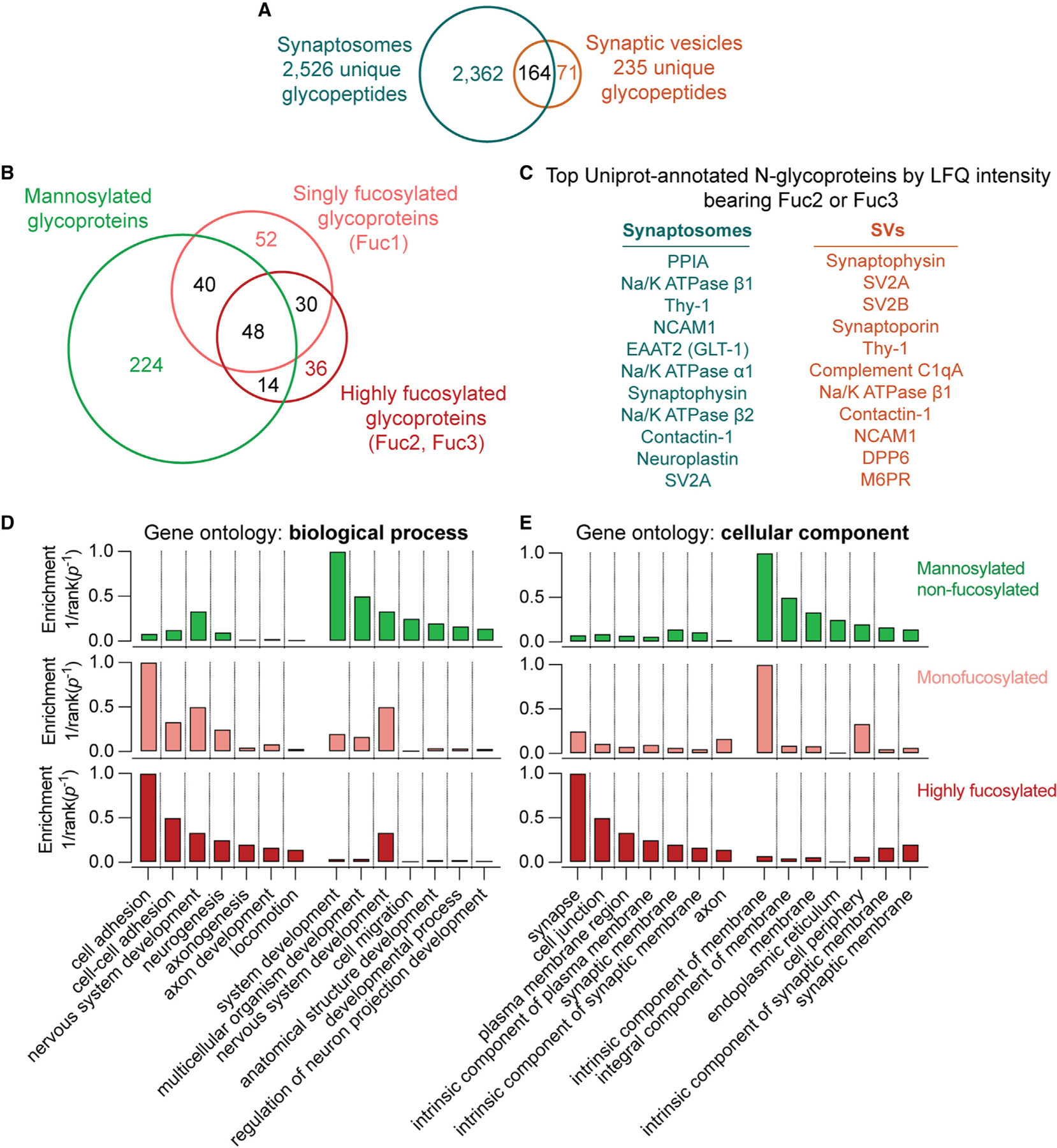
High fucosylation is characteristic of proteins at the SV and plasma membrane (A) Venn diagram of unique glycopeptides detected in synaptosomes and SVs. Approximately 70% of SV glycopeptides were also detected in synaptosome samples. (B) Venn diagram of glycoproteins bearing *N*-glycans detected in this study. While some proteins were found with only one subtype of *N*-glycosylation, many proteins contained both mannosylated and fucosylated *N*-glycans. (C) The top 11 proteins by LFQ intensity with Uniprot-annotated *N*-glycosylation sites in synaptosomes and SVs bearing antennary fucose, as defined by the presence of at least 2 fucoses. Antennary fucosylation was observed on almost all of the most abundant SV glycoproteins along with a number of cell adhesion proteins with roles in synaptic development. (D) Enrichment for Gene Ontology (GO) biological process terms in non-fucosylated, singly fucosylated, and highly fucosylated proteins. GO terms were ranked by inverse p value for enrichment, and the top 7 terms enriched in highly fucosylated or non-fucosylated proteins are shown. Non-fucosylated proteins demonstrate substantially less enrichment of cell adhesion processes. (E) As in (D) but for GO cellular component terms. Synapse- and plasma membrane-related components predominate in highly fucosylated proteins, while non-fucosylated proteins are more enriched in endoplasmic reticulum and non-specific membrane components. See also Table S4, which contains all glycoprotein glycosylation category assignments and GO biological process enrichment data used to generate this figure.

We conducted further analyses of our glycoproteomics data to better define the biological context for antennary fucosylation at central synapses. Among unique glycopeptides found in SVs, the majority (164/235) were also found in synaptosome samples (Figure 7A), consistent with the observed overlap in the proteomic contents of these samples (Figures 1E and 1F). A Venn diagram describing the distribution of all proteins with detected *N*-glycans is shown in Figure 7B. As expected, the majority of the glycoproteins with annotated glycoPSMs in this study were mannosylated (Figures 4, S2, and S3), with a relatively smaller proportion of proteins observed with singly fucosylated and highly fucosylated complex *N*-glycans (Figure 7B). In agreement with previous large-scale glycoproteomic studies of mouse brain,^39^ we observed that many proteins contained both mannosylated and fucosylated *N*-glycans (Figure 7B).

A closer examination of the identified glycopeptides in SVs and synaptosomes revealed a striking relationship among fucosylation, SV trafficking, cell adhesion, and other functions at the plasma membrane. While synaptosomes contained a broader sample of glycopeptides compared with SVs (Figure 7A), the SV proteins synaptophysin and SV2A were among the most abundant (by LFQ intensity) highly fucosylated proteins in synaptosomes (Figure 7C). Indeed, high fucosylation was observed on nearly all the most abundant SV glycoproteins, except for synaptotagmin-1 (Figures 7C, 1D, and 6). High fucosylation was also observed on cell adhesion molecules with roles in axonal pathfinding and synapse formation, including contactin-1,^66^ neuroplastin,^67–69^ and NCAM1^70^ (Figure 7). The highly abundant plasma membrane glycoprotein Thy-1,^63,64^ the plasma membrane Na^+^/K^+^-ATPase, and the plasma membrane glutamate transporter EAAT2/GLT-1^71–73^ were also among the top 10 most abundant highly fucosylated proteins in synaptosomes (Figure 7C). Of note, we also detected fucosylated *N*-glycans on peptides expected to reside in the cytoplasm for some highly abundant proteins in both SVs and synaptosomes (Table S3).

Gene Ontology (GO) analysis demonstrated that cell adhesion was among the most enriched biological process terms in highly fucosylated proteins (Figure 7D). Other highly enriched processes included several that are essential for synaptic development, including neurogenesis, axonogenesis, and chemotaxis (Figure 7D). There was substantial overlap in top-ranked GO biological process enrichment terms between singly fucosylated and highly fucosylated proteins (Figure 7D), while proteins that were not fucosylated demonstrated substantially lower enrichment of cell adhesion-related processes (Figure 7D). Correspondingly, GO cellular component analysis demonstrated a predominance of synapse- and plasma membrane-related terms among highly fucosylated proteins, while non-fucosylated proteins were enriched for ER and non-specific membrane-related cellular components (Figure 7E). All ranked GO terms are shown in Table S7. While a previous study using lectin affinity enrichment implied a connection between fucose-α(1,2)-galactose and plasma membrane-related processes in mouse olfactory bulb,^74^ we are unsure how to interpret those results given evidence that the mouse brain is largely devoid of fucose-α(1,2)-galactose and the enzymes that catalyze its addition^22,74^ (Figure S5). Despite this discrepancy, our work provides foundational evidence to inform proposed links among protein fucosylation, presynaptic membrane trafficking, and cell adhesion-related processes in the mammalian brain.^29,31,74^

## DISCUSSION

The present study demonstrates the power of combining stringent organelle purification and chemical analysis to address—and generate—specific questions in neurobiology. Our proteomics results (Figures 1 and 2) represent coverage of the SV proteome at unprecedented depth, providing a valuable resource for investigators and extending the body of evidence supporting the use of modern IP techniques for SV purification.^41^ The combination of SV-IP, glycan-focused MS, and fluorescence methods revealed that SVs bear a distinctive *N*-glycan signature marked by oligomannose and highly fucosylated glycans (Figures 4, 5, 6, and 7). We note that a prior study^39^ reported a larger number of glycopeptides using whole mouse brain, lectin affinity chromatography, offline fractionation, and AI-ETD. However, a comparison between the present study and the work of Riley et al.^39^ demonstrates the utility of our focused approach using SV protein purification and SAX-ERLIC, which represents a compromise of breadth for depth at the synapse. For example, MSFragger-Glyco analysis of the dataset from Riley et al. yields no glycoPSMs for synaptophysin or synaptoporin, and the glycoPSMs found for SV2 isoforms contain at most one fucose (Table S8). By contrast, we found 15 unique glycoforms for synaptophysin alone, many of which were highly fucosylated (Figure 6). Another prior glycoproteomic study of synaptosomes,^40^ which used a HexNAc-binding lectin to enrich brain glycopeptides and correspondingly found HexNAc to be the most common *N*-glycan, likewise did not report synaptophysin glycosylation. By comparison, truncated *N*-glycans were relatively rare in our sample (Figures S2 and S3), likely due in part to differences in enrichment method. Moreover, while previous studies have identified brain glycans with high degrees of antennary fucosylation corresponding to Lewis glycoepitopes or possibly fucose-α(1,2)-galactose,^22,29,40,54,74^ this study defines subcellular distributions of protein glycosylation at the synapse in unprecedented detail. Our work defines high fucosylation as a molecular signature of proteins at the SV and plasma membrane (Figures 4, 5, 6, and 7).

Our deep profiling of the SV *N*-glycoproteome raises several questions about the processing of *N*-glycans on proteins that undergo antennary fucosylation. Strikingly, SV proteins largely contained a single preferred site for fucosylation, while other sites were dominated by less mature mannosylated glycoforms (Figure 6). These results are consistent with our HPLC results, which demonstrate that SVs still contain a substantial proportion of mannosylated glycans despite being enriched for antennary fucosylated structures (Figure 5). Cleavage of mannose residues, and subsequent addition of GlcNAc and antennary fucose, thus appears to be site specific. The basis for this site selectivity is unclear, though we note that glycan hetereogeneity^39^ has been described in many other contexts, e.g., on the HIV envelope glycoprotein.^75^ Differential accessibility of glycan sites to glycan processing enzymes may shape glycan distributions.^76^ Because only a single glycosylation site tends to undergo maturation in each SV protein (Figure 6), it is tempting to speculate that glycan processing may be a rate-limiting step in the progress of some SV proteins through the Golgi. Examples of such glycan-dependent sorting pathways exist, most notably involving ER quality control mechanisms^5^ and mannose-6-phosphate receptors that recognize this specific glycan to recruit lysosomal proteins.^77^ While an oligomannose−, GlcNAc−, or fucose-dependent Golgi transport lectin would provide an explanation for the limited maturation of all but one glycosite, more work is needed to address this hypothesis. We note that this site-specific maturation bias was less pronounced on some highly abundant plasma membrane proteins such as Thy1 (Figure 6) and Na^+^/K^+^-ATPase subunit β2 (Table S3).^39^ Top-down analyses of glycosylation across multiple sites on intact proteins may clarify the nature of the heterogeneity observed in our bottom-up studies.

The absence of fucosylation on syt1 (Figure 6), a highly abundant SV glycoprotein, raises yet more questions. At least two potential explanations exist for this divergence from other SV glycoproteins: the luminal portion of syt1, which lacks a globular domain, may disfavor certain glycan processing steps, or syt1 may not spend enough time in the Golgi compartments containing the requisite enzymes and metabolites. We note that, unlike the case for synaptophysin and SV2, trafficking of syt1 does not require *N-*glycosylation^16^ but does require at least one C2 domain,^78^ suggesting that syt1 sorts to SVs via a distinct molecular recognition process. Unfortunately, other well-established SV glycoproteins such as VGlut2 were not covered deeply enough to confidently ascertain their fucosylation status (Table S3). Further experiments combining glycosylation site mutagenesis, enzyme manipulation, microscopy, and glycoproteomics may better define the site-specific determinants of SV protein glycosylation and their role in protein trafficking.

The enrichment of antennary fucosylated glycans on SV proteins, plasma membrane transporters, and cell adhesion proteins (Figures 4, 5, 6, and 7) is a key finding that merits further investigation. Loss of antennary fucosylation via deletion of Fut9 may drive mouse behavioral abnormalities,^29^ cellular migration deficits,^29^ and impaired neurite outgrowth.^31^ The abundance of antennary fucosylation on proteins important for cell adhesion and synaptogenesis (Figure 7) suggests that this glycan moiety may impact the biosynthesis, trafficking, or function of some of these proteins. The commonality of antennary fucosylation among SV and plasma membrane proteins (Figure 7) is consistent with the notion that SV proteins first traffic to the plasma membrane prior to sorting to SVs.^79,80^ While high fucosylation is neither necessary nor sufficient for protein trafficking to SVs (Figures 4, 6, and 7), the enrichment of the Fuc3 glycan *φ* on SVs (Figure 5) suggests that SV recycling is associated with the presentation of fucosylated glycans at high density on the presynaptic plasma membrane. At present, it is uncertain whether *φ* represents a Lewis glycoepitope, and the endogenous binding sites in the brain for antennary fucosylated glycans are, to our knowledge, unknown. Further studies examining specific links among antennary fucosylation, protein trafficking, neuronal circuits, and synaptic physiology are needed to clarify the functional roles of specific *N*-glycans.

Our findings are particularly striking given the recent identification of *FUT9* as a gene locus linked to schizophrenia.^33^ Abnormal glycosylation of the antennary fucose-bearing proteins EAAT2/GLT-1^81^ and GABA_A_ receptor subunits (Tables S3 and S7)^82^ has been described in schizophrenia post-mortem brain studies. Altered fucosyltransferase enzyme levels have also been described in similar work.^83^ Given that the specific brain functions of antennary fucosylation remain undefined, the potential links between antennary fucosylation and schizophrenia are numerous. For example, inefficient protein trafficking to axonal projections would broadly inhibit neurite outgrowth and neurotransmission, while reduced efficacy of cell-cell adhesion might reduce the stability of neuronal circuits. We note that fucose and fucosylated glycans are reportedly detectable in brain by magnetic resonance imaging (MRI) spectroscopy,^84^ and their study may thus yield insights into the biology of schizophrenia and other developmental processes across the human lifespan.

Finally, we emphasize the need for further investigation into potential links among protein glycosylation, the innate immune system, and synaptic pruning. Several studies have demonstrated a role for complement proteins in both synaptic pruning^85,86^ and schizophrenia,^87^ and lectins can directly activate the complement cascade.^88^ Among the best-known complement-activating lectins is mannose-binding lectin, which is activated by mannosylated glycans typically found on microorganisms but not on circulating glycoproteins.^88^ Mannose-binding lectin is a well-known mediator of inflammatory brain injury after ischemia or trauma.^89,90^ Strikingly, mannosylated glycans are common on synaptic glycoproteins^22^ (Figures 4, 5, 6, and 7), and studies of experimental autoimmune encephalitis suggest that d-mannose may inhibit phagocytosis by activated microglia.^91^ Restricting neuronal N-glycans to oligomannose types by enzyme knock-out causes rapid neurological decline and neuronal apoptosis after birth, though the pathophysiology of this phenotype is unclear.^25^ Roles for neuronal *N*-glycans in modulating complement activation and synaptic pruning, particularly during development or periods of neuroinflammatory stress,^46,92^ remain to be determined.

### Limitations of the study

Sialic acid, a biologically important sugar that commonly decorates *N*-glycans on plasma proteins, was omitted from our analysis. While we detected sialylated glycopeptides (Table S3; Figures S2 and S3), these were omitted due to a concern that these may represent glycopeptides decorated with both *N*-and *O*-glycans.^22^ We note that SVs may also contain a unique subset of *O-*linked glycans, which were not characterized in this study. Another limitation of this study is the undefined identity of the highly fucosylated, SV-enriched *N*-glycan *φ* (Figure 5). Finally, as with other LC-MS glycoproteomic studies,^39,40^ we also do not address the large, poly-anionic proteoglycans associated with the extracellular matrix and perineuronal nets,^51,93^ which are comparatively poor analytes for positive-mode MS. Future application of negative ionization and fragmentation approaches, such as negative electron transfer dissociation (e.g., AI-NETD),^94^ may enable the characterization of glycans not detected in the present study.

## STAR★METHODS

### RESOURCE AVAILABILITY

#### Lead contact

Further information and requests for resources and reagents should be directed to and will be fulfilled by the lead contact, Mazdak Bradberry (mazdak.bradberry@nyspi.columbia.edu).

#### Materials availability

This study did not generate new unique reagents.

#### Data and code availability

- Raw LC-MS data are publicly available online via the MassIVE repository at https://doi.org/10.25345/C5TB0Z526. Relevant MSFragger output tables are included in Tables S1 and S2.
- All original R scripts used to process the MSFragger output have been deposited at Zenodo and are available at https://doi.org/10.5281/zenodo.7659070
- Any additional information required to re-analyze the data reported in this paper is available from the lead contact upon request.

### EXPERIMENTAL MODEL AND SUBJECT DETAILS

C57B/6J mice of either sex between 15 and 20 days of age were used for all experiments. Juvenile mice were used with the objective of capturing both immature and mature glycosylation patterns.^20,24^ All work was conducted according to protocols approved by the University of Wisconsin Institutional Animal Care and Use Committee.

### METHOD DETAILS

#### Antibody and bead preparation

Anti-SV2 mAb (SV2, DSHB) and anti-syt1 mAb (mAb 48, DSHB) were purified from ascites stocks generated prior to 2010 using protein G chromatography (Protein G Sepharose Fast Flow, Cytiva) and dialyzed extensively against phosphate-buffered saline (140 mM NaCl, 10 mM sodium phosphate buffer, pH 7.4). Single-use aliquots (200 μL, 300 μg) of mAb in PBS were kept frozen at −80°C. Dynabeads M-270 Epoxy (14302D, Thermo Fisher) were coupled to mAb according to published procedures.^41^ For each coupling reaction, 10 mg of Dynabeads stored in DMF were collected with a magnetic stand and resuspended in 200 μL borate buffer (100 mM boric acid-NaOH, pH 8.5). To this suspension was added 200 μL mAb solution, followed by 200 μL 3 M ammonium sulfate in borate buffer, with mixing by pipetting up and down after each addition. This coupling mixture was incubated with rotation at 37°C overnight. The beads were then collected with a magnetic stand, the supernatant was discarded, and the beads were washed six times by trituration in 1 mL wash buffer followed by collection with the magnetic stand. The wash buffers were 500 mM NaCl, 50 mM ammonium acetate pH 4.5 and 500 mM NaCl, 50 mM Tris-HCl pH 8.0, used in an alternating manner (i.e., one buffer followed by the other, for three cycles total). The beads were then resuspended in 1 mL KPBS (145 mM KCl, 10 mM potassium phosphate buffer pH 7.4), transferred to a fresh tube, and collected on a magnetic stand. The supernatant was removed and the beads were resuspended at 30 mg/mL using 300 μL KPBS. Beads stored in this manner at 4°C remained effective for at least 2 months.

#### SV preparation

All buffers, tubes, and centrifuge rotors were cooled to 0–2°C prior to use. One or two mice were anesthetized with isoflurane, euthanized, and the brains including cerebellum and brain stem were rapidly removed. Each brain was placed in a tight-fitting Teflon-glass Dounce homogenizer with 3.8 mL ice-cold potassium homogenization buffer (125 mM KCl, 25 mM potassium phosphate buffer, 5 mM EGTA, pH 7.4) containing protease inhibitors (cOmplete mini EDTA-free, Roche, 1 tablet/10 mL buffer) and homogenized with ten strokes using an overhead mixer rotating at 900 RPM. The homogenate was then centrifuged at 35,000 x g for 20 min at 2°C. During centrifugation, 5 mg α-SV2 Dynabeads per brain were washed in KPBS and resuspended in two 2-mL microcentrifuge tubes (2.5 mg beads in 100 μL in each tube). The supernatant from each brain homogenate was added to two tubes containing Dynabeads (1.9 mL/tube), and the tubes were placed inside 50-mL conical tubes packed with ice and incubated with rotation for 25 min in a cold room. The supernatants were then discarded and each 2.5 mg portion of beads, corresponding to SVs from ½ mouse brain, was washed 3 times by gentle trituration in 1 mL ice-cold KPBS followed by collection on a magnetic stand. The beads were then resuspended and transferred to a fresh tube using KPBS, with beads bearing SVs from the same brain combined into the same tube (5 mg/tube), and the supernatant was removed. For proteomics and glycoproteomics studies, the beads were eluted using 50 μL 2% SDS containing 25 mM Tris-HCl pH 8.0 with heating to 50°C for 5 min, and the eluates were frozen at −80°C prior to use. For HPLC studies, the beads were eluted using 45 μl 0.5% SDS with heating to 50°C for 5 min, followed by a second elution with 45 μL 2% n-β-dodecylmaltoside (DDM) (Gold Biotechnologies) for 5 min at room temperature. These eluates were combined and 2 μL 1 M triethylammonium bicarbonate (TEAB) was added prior to storage at −80°C.

#### Synaptosome preparation

Synaptosomes were prepared by removing and homogenizing brains as above except that the homogenization buffer contained 125 mM NaCl, 25 mM HEPES-NaOH, and 5 mM EGTA, pH 7.4. The brain homogenate was centrifuged at 3,500 x g for 2 min, the pellet was discarded, and the supernatant transferred into two 2-mL microcentrifuge tubes and centrifuged for 12 min at 14,000 x g at 4°C. The supernatant was discarded and the pellet was resuspended in 1530 μL 50 mM TEAB per 2-mL tube. 90 μL of 10% SDS and 180 μL 10% DDM (0.5% SDS, 1% DDM final) were added to each tube, which was incubated for 1 h with rotation at 4°C prior to aliquoting and freezing at −80°C.

#### Immunopurification of SV2 and syt1

SV2 and syt1 were immunoprecipitated from synaptosomes prepared as above but resuspended in 1.6 mL synaptosome homogenization buffer followed by the addition of 180 μL 10% DDM (~1% DDM final) per one-half brain. The samples were incubated with rotation for 1 h at 4°C followed by pelleting of insoluble material by centrifugation at 20,000 x g for 20 min at 4°C. The supernatants were transferred to new tubes, 3 mg α-syt1 or α-SV2 Dynabeads were added, and the tubes were incubated with rotation for 30 min. The beads were then washed with cold synaptosome resuspension buffer (4 × 1 mL) and eluted with 50 μL 2% SDS containing 25 mM Tris-HCl pH 8.0 with heating to 50°C for 5 min. Eluates were stored frozen at −80°C prior to use.

#### Tryptic peptide preparation

For immunopurified SV, syt1, and SV2 samples, ~60 μL bead eluate in SDS was combined with dithiothreitol (DTT, 100 mM freshly prepared aqueous stock solution) for a final concentration of 5 mM DTT and incubated at 50°C for 25 min. Iodoacetamide (200 mM freshly prepared aqueous stock solution) was added to a final concentration of 15 mM and the reaction incubated in the dark at room temperature for 30 min. More DTT was then added (26 mM final concentration) to quench iodoacetamide. Dynabeads M-270 carboxylic acid (14305D, Thermo Fisher Scientific) were then added (2 μg/μL final concentration) and the tubes were mixed well, followed by the addition of 1 volume of absolute ethanol and brief incubation on a thermomixer (5′, 1000 RPM, 23°C) to drive protein adsorption to the beads. The beads were washed three times with 200 μL 80% ethanol and transferred to a fresh tube with the final wash. The supernatant was removed and tryptic peptides were eluted from the beads by overnight digestion in 50 μL trypsin solution (V5111, Promega, 0.01 μg/μL in 100 mM ammonium bicarbonate) with shaking in a thermomixer (1000 RPM, 37°C). Eluates from this step were used directly for LC-MS or subject to glycopeptide enrichment (*vide i****n****fra*). For synaptosomes, a similar procedure was followed but scaled up 10-fold, using 500 μL synaptosomal lysate as input, washes of 3 × 1 mL 80% ethanol, and elution in 250 μL trypsin solution.

#### Glycopeptide enrichment

Glycopeptides were enriched by strong anion exchange-electrostatic repulsion chromatography (SAX-ERLIC) according to recently published procedures.^58^ Strong anion exchange columns (SOLA SAX 10 mg, Thermo Fisher) were washed with acetonitrile (3 × 1 mL), 100 mM triethylammonium acetate in water (3 × 1 mL), 1% trifluoroacetic acid (TFA) in water (3 × 1 mL), and 1% TFA in 95:5 MeCN:H_2_O. Tryptic peptides (100 μL for synaptosome samples, 50 μL for SV samples) were brought up to 95:5 MeCN:H_2_O with the addition of 19 volumes of MeCN and applied to the column twice. The column was then washed with 95:5 MeCN:H_2_O containing 1% TFA (6 × 1 mL). Glycopeptides were eluted with 50:50 MeCN:H_2_O containing 1% TFA (850μL + 500 μL) followed by 95:5 H_2_O:MeCN containing 1% TFA (850μL + 500 μL). The eluates were dried in a speedvac and stored at −80°C. For each sample, 30 μL 0.2% formic acid in water was used to redissolve all dried eluates prior to LC-MS analysis.

#### nLC-MS/MS

Data were collected using two systems, each comprising a hybrid Orbitrap mass spectrometer (Orbitrap Eclipse or Oribtrap Fusion Lumos, Thermo) interfaced to a nanoflow HPLC system (UltiMate 3000, Dionex) via a nanospray ionization source (Nanospray Flex, Thermo). Samples were separated using a column with integrated spray tip (PicoTip SIS, 25 cm long, 75 μm I.D.) packed in-house with C18 particles (BEH C18 1.7 μm, Waters) at ultra-high pressure^95^ and held at 50°C using a custom-built column heater. For standard proteomics experiments (Figures 1 and 2), mobile phase A was 0.1% formic acid in H_2_O, mobile phase B was 0.1% formic acid in 80:20 MeCN:H_2_O, and peptides were separated using a 2-h gradient as follows: 0–17 min, 0–7% B; 17–102 min, 7–50% B; 102–104 min, 50–100% B; 104–108 min, 100% B; 108–110 min, 100–0% B; 110–120 min, 0% B. The flow rate was 310 nL/min, the spray voltage was 2 kV and the injection volume was 3 μL. For proteomics runs (Figures 1 and 2), MS^1^ and MS^2^ scans were acquired in positive mode in the Orbitrap, and the following settings were used for MS^1^ spectra: resolution, 120,000; scan range, 400–1600 *m/z*; maximum injection time, 50 ms; AGC target, 400,000; normalized AGC target, 100%. MS^2^ spectra were acquired with the following settings: resolution, 30,000; scan range, 150–1800 *m/z*; maximum injection time, 60 ms; AGC target, 50,000; normalized AGC target, 100%; HCD collision energy, 30%. MS^1^ peaks were filtered based on the following criteria for fragmentation: charge state, 2–8; maximum intensity, 1E20, minimum intensity, 50,000. Monoisotopic precursor selection was used in peptide mode, and MS^1^ peaks were dynamically excluded for 20 s with a 20-ppm mass tolerance after being selected for fragmentation. For glycoproteomics runs (Figures 3, 4 and 6), the same LC gradient and spray voltage were used for most experiments (see below). Glycoproteomics MS^1^ scans were obtained in positive mode every 3 s with the following settings: resolution, 120,000; scan range, 350–1800 *m/z*; maximum injection time, 50 ms; AGC target, 400,000; normalized AGC, target 100%. Peaks were selected for MS^2^ fragmentation from charge states 2–8 with dynamic exclusion in a ±10 ppm window for 60 s after a single detection, with monoisotopic precursor selection enabled. For MS^2^ scans, precursors were fragmented with higher-energy collisional dissociation (HCD) with HCD energy 36%; resolution, 30,000; maximum injection time, 60 ms; AGC target, 50,000; normalized AGC target, 100%. A second fragmentation scan of the same precursor was triggered if one of the top 20 most abundant ions in the first MS^2^ spectrum was one of the following: *m/z* 204.0867, 138.0545, 366.1396, 274.0921, 292.1027, 126.055, 144.0655, 168.0654, 186.076. This second, triggered scan activated ion with either used stepped HCD (sceHCD) or activated ion-electron transfer dissociation (AI-ETD). SV samples were analyzed using sceHCD, while both sceHCD and AI-ETD were employed for synaptosome samples given their greater complexity and abundance of material for analysis. For AI-ETD, an Orbitrap Fusion Lumos mass spectrometer (Thermo Fisher Scientific, San Jose, CA) was retrofitted with a 60 W CO2 laser to allow for photoactivation.^55,56^ For sceHCD, energies of 20%, 35%, and 50% were used (Riley et al., 2020). For AI-ETD, calibrated ETD reaction parameters were used, and the laser was operated at either 7% or 10% maximum power. All sceHCD and AI-ETD scans used the following parameters: scan range, 120–4000 *m/z*; maximum injection time, 200 ms; AGC target, 50,000; normalized AGC target, 100%. For non-enriched synaptic vesicle samples, we also included glycoproteomics data from a set of runs using an alternative LC solvent system (four biological replicates total). For these runs, mobile phase A was 0.2% formic acid in H_2_O, mobile phase B was 90:10 isopropanol:acetonitrile with 0.2% formic acid and 5 mM ammonium formate, and peptides were separated using a 2-h gradient as follows: 0–13 min, 0% B; 13–18 min, 0–3% B; 18–88 min, 3–22% B; 88–100 min, 22–70% B; 100–101 min, 70–85% B; 101–105 min, 85% B; 105–106 min, 85–0% B; 106–120 min, 0% B. In this method the column was kept at 60°C and the flow rate was 225 nL/min except during periods of high %B (88–106 min), when it was reduced to 200 nL/min.

#### LC-MS/MS data analysis

Raw files from LC-MS runs were analyzed using the FragPipe software suite (v.17.1) with further processing of FragPipe output performed in R. All R scripts used are available via Github at https://github.com/mazbradberry/public/tree/glycoproteomics. For proteomics experiments with label-free quantification (LFQ), SV and synaptosome experiments were analyzed separately. Spectra were searched with MSFragger (v.3.4)^59^ using a mouse proteome database downloaded from Uniprot on 11 October 2021 and the following settings: precursor mass tolerance, ±20 ppm; fragment mass tolerance, ±20 ppm, mass calibration and parameter optimization enabled; isotope error, 0/1/2; enzymatic cleavage, strict trypsin with up to 2 missed cleavages; peptide length, 7–50; peptide mass range, 500–5000 Da. Methionine oxidation and N-terminal acetylation were allowed as variable modifications and cysteine carbamidomethylation was included as a fixed modification. Validation was performed with PeptideProphet using closed search defaults for peptides and ProteinProphet for proteins. LFQ was performed with IonQuant^96^ with match-between-runs, normalization, and MaxLFQ enabled. The following settings were used: feature detection m/z tolerance, ±10 ppm; feature detection RT tolerance, 0.4 min; match between runs (MBR) tolerance 5 min, MBR ion FDR, 0.01; MBR peptide and protein FDR, 1; top 3 ions used for quantification with a minimum frequency of 0.5 and detection in at least 1 experiment. For glycoproteomics experiments, runs were grouped by sample type (SV, SV with enrichment, or synaptosomes with enrichment) prior to analysis. The same mouse protein database was used and spectra were searched with MSFragger (v.3.4) using glyco mode^38,60^ with the following settings: precursor mass tolerance, ±20 ppm; fragment mass tolerance, ±20 ppm, mass calibration and parameter optimization enabled; isotope error, 0/1/2; enzymatic cleavage, strict trypsin with up to 2 missed cleavages; peptide length, 7–50; peptide mass range, 400–5000 Da. Methionine oxidation and N-terminal acetylation were allowed as variable modifications and cysteine carbamidomethylation included as a fixed modification. The default 183 mass offsets corresponding to possible *N*-glycan compositions were included and restricted to asparagine residues. Labile modification search mode was set to nglycan and diagnostic Y ion and fragment masses were left as defaults. PTM-Shepherd was enabled, diagnostic ion search was enabled with default settings, and glycans were assigned in N-glycan mode with an FDR of 0.025, mass tolerance of ±50 ppm, and isotope error range of −1 to 3. PeptideProphet and ProteinProphet were used for peptide and protein validation, respectively, with default settings. The same parameters were used for glycoproteomic studies of immunoprecipitated SV2 and syt1. Glycopeptide spectral matches (GlycoPSMs) were obtained from the psm output file, filtered for Q-values of ≤0.025, and annotated using the table shown in Table S3, with sialic acid-containing compositions excluded fron analysis due to the possibility that they represent combinations of *N*-and *O*-glycosylation on the same peptide.^22^ Tetra-fucosylated glycans and compositions tentatively identified as singly fucosylated oligomannose glycans were also detected but were omitted from analysis given their uncertain identification and small contribution (<5%) to the total glycan pool. Relative frequencies of unique annotated and included *N*-glycans, along with glycoprotein LFQ intensity quintiles (Figure 4), were determined using R. Glycoprotein abundance quintiles were determined using only glycoproteins; for example, 80–100% indicates the most abundant 20% of glycoproteins, not glycoproteins in the top 20% of all proteins. For GO analysis (Figure 7), gene lists were extracted from annotated glycoPSM tables and subjected to biological process or cellular component GO term search (geneontology.org, accessed 25 April 2022).^97,98^ Organelle markers (Figure 2) were determined using targets available in the Thermo Fisher antibody catalog (https://www.thermofisher.com/us/en/home/life-science/antibodies/primary-antibodies/organelle-marker-antibodies.html, accessed 28 November 2022) with the addition of widely used neuronal surface and SV markers.

#### *N*-glycan release and labeling

For synaptosome samples, 50 μL synaptosomal lysate in 1% DDM and 0.5% SDS was combined with 50 μL 2% DDM and 5 μL1 M DTT and heated to 50°C for 15 min. For SV samples, 100 μL of SDS-DDM eluate (0.25% SDS, 1% DDM final) was combined with 5 μL 1M DTT and heated to 50°C for 15 min. For each sample, 1 μL PNGase F solution (P0708, NEB) was then added, and the mixture was incubated at 37°C for 2 h. Each sample was allowed to cool to room temperature, combined with 50 μL of freshly prepared procainamide solution (40 mg/mL in 70:30 DMSO:acetic acid) and incubated on ice for 5 min. Samples were then centrifuged (20,000 x g, 10 min, 4°C) and the supernatants (150 μL) transferred to fresh PCR tubes. 10 μL of sodium cyanoborohydride solution (5 M in 1 M NaOH, Sigma 296945) was added to each reaction, which was then incubated at 65°C for 2 h in a fume hood using a miniature PCR block. All steps involving sodium cyanoborohydride, including sample cleanup, were carried out in a fume hood. The samples were then combined with 20 volumes of MeCN (i.e., 75 μL sample was added to 1.5 mL MeCN) in 2-mL microcentrifuge tubes and subjected to cleanup by solid-phase extraction using a vacuum manifold. For each sample, a solid-phase extraction cartridge (OASIS HLB 30 mg, Waters) was equilibrated with 1 mL 95:5 MeCN:H_2_O, and the sample (~3.2 mL) was applied. The cartridge was washed (2 × 1 mL 95:5 MeCN:H_2_O) and procainamide-labeled glycans eluted with 500 μL 50:50 MeCN:H_2_O. The eluates were dried using a speedvac, resuspended in Milli-Q water (200 μL for synaptosome samples, 40 μL for SV samples), and centrifuged to remove insoluble material.

#### Exoglycosidase digestion and HPLC sample preparation

4 μL procainamide-labeled glycans were combined with 1 μL 10x sodium acetate – Ca^2+^ buffer (Glycobuffer 1, New England Biolabs), 1 μL 10x BSA (diluted 1:10 with Milli-Q water from a 100X stock, New England Biolabs), and 1 μL of each enzyme used as described in Figures 5 and S5. Enzymes used included α(1–2,3,6) mannosidase (P0768, New England Biolabs [NEB]), α(1–3,4) fucosidase (P0769, NEB), α(1–2) fucosidase (P0724, NEB), α(1–2,4,6) fucosidase O (P0749, NEB); β(1–3,4) galactosidase (P0746, NEB), and β-GlcNA-case S (P0744, NEB). In reactions containing α-mannosidase and for all experiments shown in Figure 5, 1.2 μL 10 mM zinc chloride (New England Biolabs) was included. Each reaction was brought up to 10 μL with Milli-Q water and incubated overnight at 37°C ina PCR block. 15 μL of HPLC-grade acetonitrile was then added, the samples were centrifuged (20,000 x g, 10 min, 4°C), and the supernatants stored on ice until HPLC analysis.

#### HILIC-HPLC analysis

An Agilent HPLC system (Infinity 1260 Bio-inert) equipped with a fluorescence detector (Agilent 1260 FLD Spectra, 310 nm excitation, 370 nm emission), amide HILIC column (Agilent Glycan Mapping, 2.1 mm × 150 mm, 2.7 μm particle size) and manual injector was used for analysis of procainamide-labeled glycans. 10 μL glycan digest was injected to overfill a 5 μL home-cut sample loop and the column was kept at 40°C. Mobile phase A was 100 mM ammonium formate, pH 4.4, mobile phase B was 100% acetonitrile. Samples were separated with a 90-min gradient as follows: 0–60 min, 75–62.5% B; 60–62 min, 62.5–15% B; 62–82 min, 15% B; 82–85 min, 15–75% B; 85–90 min, 75% B. The flow rate was 0.25 mL/min except during periods of lower % B (62–85 min), when it was reduced to 0.175 mL/min 30 min was allowed between runs for re-equilibration. Peak areas were determined by automatic integration in Agilent ChemStation software with the following settings: tangent skim mode, new exponential; tail peak skim height ratio, 5.00; front peak ski height ratio, 5.00; skim valley ratio, 20.00; baseline correction, advanced; peak to valley ratio, 500. Peaks eluting between 19 and 60 min were considered for analysis.

#### SDS-PAGE and immunoblot

Synaptosome and SV samples were combined with 4X SDS sample buffer containing DTT and heated to 50°C for 15 min except as shown in Figure S6. 10 μL of prepared sample containing 6.7 μL synaptosome lysate or 1–1.5 μL SV eluate was subjected to SDS-PAGE on 4–20% gradient gels (Criterion TGX, Bio-Rad) and transferred to a PVDF membrane using a semi-dry blotting apparatus. Blots were blocked using TBS-T (150 mM NaCl, 10 mM Tris-HCl pH 7.4, 0.1% Tween 20) containing 5% nonfat dry milk and incubated overnight at 4°C with primary antibody in TBS-T containing 1% nonfat dry milk. Antibodies for immunoblot included guinea pig anti-synaptophysin (101 004, Synaptic Systems, 1:1000 dilution of a 0.5 mg/mL stock) or mouse monoclonal anti-SV2 (SV2, DSHB, 1:1,000 dilution of a 1.2 mg/mL stock purified from ascites). Blots were washed in TBS-T, and HRP-labeled secondary antibodies were used for detection.

### QUANTIFICATION AND STATISTICAL ANALYSIS

In Figures 1C and 1D, normalized LFQ values from three biological replicates comprising three technical replicates for each condition (i.e., three mice earch for SVs and synaptosomes) are plotted with error bars representing standard deviation. In Figure 1F, each point represents the average LFQ intensity for a protein that was observed in all three replicates for both sample type, and correlation between the datasets was assessed via the nonparametric Spearman’s correlation test (shown in figure) and by linear fitting. The linear fit and 90% prediction bands are shown on the plot. In Figure 2, proteins are shown if they are captured in at least two replicates, and bars depict 95% confidence intervals of the mean. Larger confidence intervals reflect greater uncertainty for proteins quantified in fewer replicates. In Figure 4, all glycopeptides detected in at least one of three biological replicates comprising six technical replicates (synaptosomes, sceHCD and AI-ETD) or four biological replicates comprising seven technical replicates (SVs, enriched and non-enriched) are shown. The number of unique highly fucosylated glycopeptides across LFQ quintiles for SVs and synaptosomes was compared by defining each highly fucosylated glycopeptide as a datapoint with a value corresponding to its percentile (0, 20, 40, 60, 80) and comparing the two sets with a Mann-Whitney test. In Figure 5, peak areas from four biological replicates (four mice for synaptosomes, four mice for synaptic vesicles) were quantified as described above and compared by Mann-Whitney test where indicated. In Figure 7, GO analysis was depicted by ranking the p values for each GO category from small (most enriched) to large (least enriched), then plotting the inverse of the rank.

## Supplementary Material

1

3

4

5

6

7

8

9

10

## Figures and Tables

**Table:** KEY RESOURCES TABLE

REAGENT or RESOURCE	SOURCE	IDENTIFIER
Antibodies		

SV2 mAb	DSHB	SV2 (RRID:AB_2315387)
Syt1 mAb	DSHB	mAb 48 (RRID:AB_2199314)
Guinea pig anti-synaptophysin	Synaptic Systems	104 211 (RRID:AB_1210382)
Goat anti-mouse HRP-conjugated secondary Ab	Bio-Rad	1706516
Goat anti-guinea pig HRP-conjugated secondary Ab	Thermo Fisher	A18769

Chemicals, peptides, and recombinant proteins		

Protein G Sepharose fast flow	Cytiva	17061801
Dynabeads M-270 epoxy	Thermo Fisher	14302D
cOmplete mini, EDTA-free	Sigma (Roche)	04693159001
Dynabeads M-270 carboxylic acid	Thermo Fisher	14305D
Trypsin	Promega	V5111
SOLA SAX 10 mg cartridge	Thermo Fisher	60109–003
PNGase F	NEB	P0708
Procainamide	Sigma	SML2088
Sodium cyanoborohydride, 5 M in 1 M NaOH	Sigma	296945
OASIS HLB 30 mg cartridge	Waters	WAT094225
α(1–2,3,6) mannosidase	NEB	P0768
α(1–3,4) fucosidase	NEB	P0769
α(1–2) fucosidase	NEB	P0724
α(1–2,4,6) fucosidase O	NEB	P0749
β(1–3,4) galactosidase	NEB	P0746
β-GlcNAcase S	NEB	P0744

Deposited data		

LC-MS raw data deposited at MassIVE	This paper	https://doi.org/10.25345/C5TB0Z526

Experimental models: Organisms/strains		

Mouse: C57B6/J	Jackson Laboratory	000664 (RRID:IMSR_JAX:000664)

Software and algorithms		

FragPipe	Nesvizhskii Lab	v. 17.1
Agilent ChemStation	Agilent	LTS 01.11
R/Rstudio	CRAN/Posit	v. 2021.09.0
R scripts used in this paper	This paper	https://doi.org/10.5281/zenodo.7659070
The Gene Ontology resource	Geneontology.org	v. 2023–01-01
Prism	GraphPad	v. 9.3.1

## Highlights

- Deep coverage of the ***N***-glycoproteome of brain synapses and synaptic vesicles
- Enrichment of a highly fucosylated ***N***-glycan in synaptic vesicles
- High fucosylation is characteristic of plasma membrane and SV ***N***-glycans in mouse brain
