# Supplementary material for: N-glycoproteomics of brain synapses and synaptic vesicles
Source: Cell Rep. Author manuscript; Available in PMC 2023 Oct 23. (PMC10560701; doi:10.1016/j.celrep.2023.112368)
Supplement: 1 [file NIHMS1895595-supplement-1.pdf]

Cell Reports, Volume *42*

Supplemental information

N-glycoproteomics of brain synapses

and synaptic vesicles

Mazdak M. Bradberry, Trenton M. Peters-Clarke, Evgenia Shishkova, Edwin R. Chapman, and Joshua J. Coon

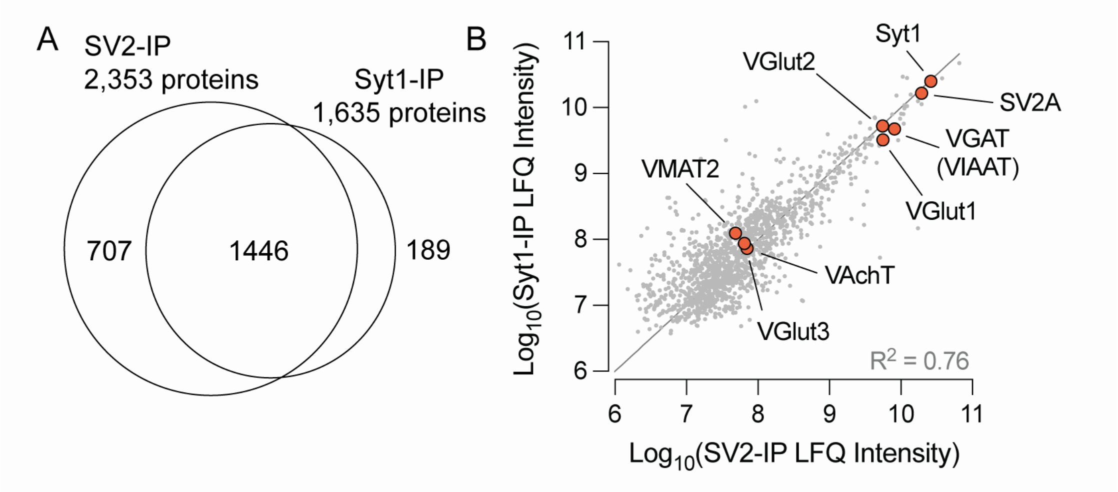

Supplementary Figure S1. Comparison between SV2-IP and Syt1-IP SV proteomes, related to Figure 1. SV2-IP LFQ data from this study were compared to recently published results using similar procedures with an anti-syt1 mAb (Bradberry et al., *J. Neurosci* 2022). (A) Venn diagram of proteins detected in SV2-IP and syt1-IP experiments. SV2-IP samples contain 88% of the proteins detected in Syt1-IP samples. (B) LFQ data from syt1-IP and SV2-IP experiments were plotted against one another. Each dot represents a protein quantified in all replicates in all experiments (1,371 proteins total). The line corresponding to $y = x$ is plotted, and R^2 for the correlation between datasets is shown. Both IP approaches yield a sample of SVs containing abundant syt1 and SV2 with markers demonstrating similar amounts of high-abundance (VGlut1/2, VGAT) and low-abundance (VAchT, VMAT) vesicle types.

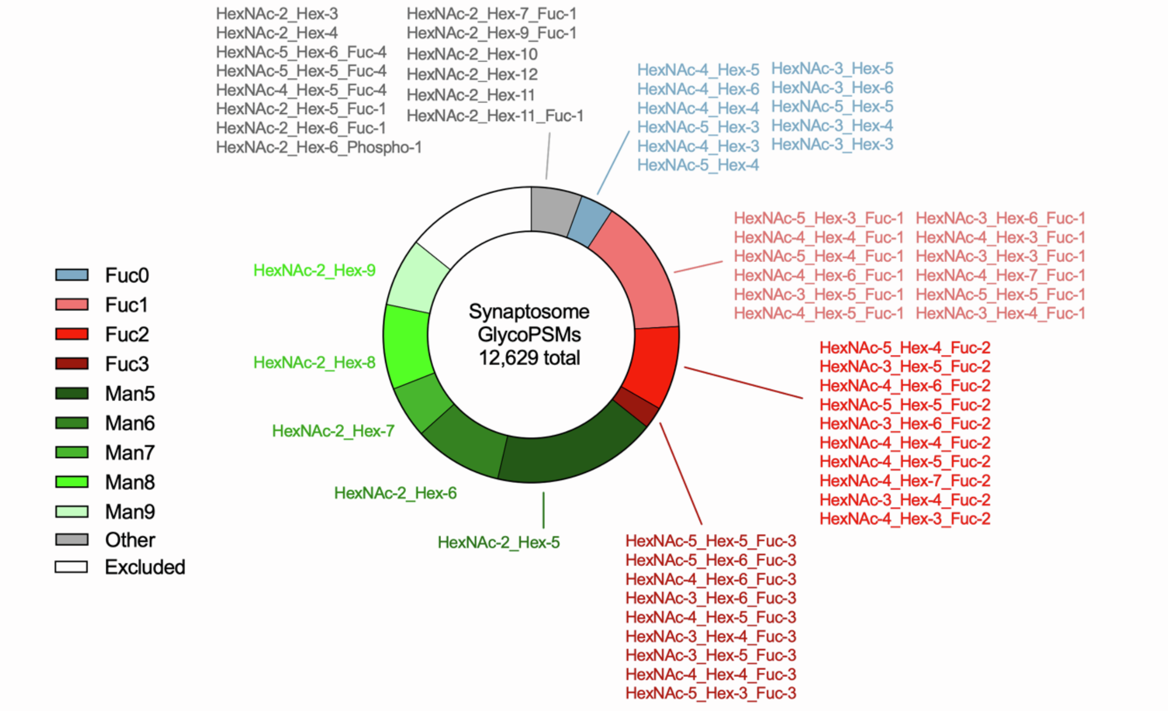

Excluded glycan compositions

HexNAc-1
HexNAc-1_Fuc-1
HexNAc-2
HexNAc-2_Fuc-1
HexNAc-2_Hex-1
HexNAc-2_Hex-1_Fuc-1
HexNAc-2_Hex-2
HexNAc-2_Hex-2_Fuc-1
HexNAc-2_Hex-3_Fuc-1
HexNAc-2_Hex-4_Fuc-1
HexNAc-3_Hex-4_NeuAc-1
HexNAc-3_Hex-4_NeuGc-2
HexNAc-3_Hex-5_Fuc-1_NeuAc-1
HexNAc-3_Hex-5_Fuc-1_NeuAc-2
HexNAc-3_Hex-5_NeuAc-1
HexNAc-3_Hex-6_Fuc-1_NeuAc-1
HexNAc-3_Hex-6_NeuAc-1
HexNAc-4_Hex-4_Fuc-1_NeuAc-1
HexNAc-4_Hex-4_Fuc-1_NeuGc-2
HexNAc-4_Hex-4_NeuAc-1
HexNAc-4_Hex-5_Fuc-1_NeuAc-1
HexNAc-4_Hex-5_Fuc-1_NeuAc-2
HexNAc-4_Hex-5_Fuc-1_NeuAc-4
HexNAc-4_Hex-5_NeuAc-1
HexNAc-4_Hex-5_NeuAc-2
HexNAc-4_Hex-5_NeuAc-3
HexNAc-4_Hex-6_Fuc-1_NeuAc-1
HexNAc-4_Hex-6_NeuAc-1
HexNAc-4_Hex-7
HexNAc-4_Hex-7_NeuAc-1
HexNAc-5_Hex-3_Fuc-1_NeuAc-1
HexNAc-5_Hex-3_NeuAc-1
HexNAc-5_Hex-4_Fuc-1_NeuAc-1
HexNAc-5_Hex-4_Fuc-1_NeuAc-2
HexNAc-5_Hex-4_NeuAc-1
HexNAc-5_Hex-4_NeuAc-2
HexNAc-5_Hex-5_Fuc-1_NeuAc-1
HexNAc-5_Hex-5_NeuAc-1
HexNAc-5_Hex-5_NeuAc-2
HexNAc-5_Hex-6
HexNAc-5_Hex-6_Fuc-1_NeuAc-1
HexNAc-5_Hex-6_Fuc-1_NeuAc-2
HexNAc-5_Hex-6_Fuc-1_NeuAc-3
HexNAc-5_Hex-6_NeuAc-2
HexNAc-5_Hex-6_NeuAc-3
HexNAc-5_Hex-6_NeuAc-4
HexNAc-5_Hex-7
HexNAc-5_Hex-8
HexNAc-5_Hex-9_Fuc-1
HexNAc-6_Hex-3
HexNAc-6_Hex-3_Fuc-1
HexNAc-6_Hex-3_Fuc-1_NeuAc-2
HexNAc-6_Hex-3_Fuc-2
HexNAc-6_Hex-3_Fuc-3
HexNAc-6_Hex-4
HexNAc-6_Hex-4_Fuc-1
HexNAc-6_Hex-4_Fuc-2
HexNAc-6_Hex-4_NeuAc-1
HexNAc-6_Hex-5
HexNAc-6_Hex-5_Fuc-1
HexNAc-6_Hex-5_Fuc-1_NeuAc-1
HexNAc-6_Hex-5_Fuc-1_NeuAc-2
HexNAc-6_Hex-5_Fuc-1_NeuAc-3
HexNAc-6_Hex-5_Fuc-2
HexNAc-6_Hex-5_Fuc-3
HexNAc-6_Hex-5_Fuc-4
HexNAc-6_Hex-5_NeuAc-1
HexNAc-6_Hex-5_NeuAc-2
HexNAc-6_Hex-6
HexNAc-6_Hex-6_Fuc-1_NeuAc-1
HexNAc-6_Hex-6_Fuc-1_NeuAc-2
HexNAc-6_Hex-6_Fuc-2_NeuGc-3
HexNAc-6_Hex-6_Fuc-3
HexNAc-6_Hex-6_Fuc-4
HexNAc-6_Hex-6_NeuAc-1
HexNAc-6_Hex-6_NeuAc-1_NeuGc-3
HexNAc-6_Hex-6_NeuAc-2
HexNAc-6_Hex-6_NeuAc-3
HexNAc-6_Hex-7
HexNAc-6_Hex-7_Fuc-1
HexNAc-6_Hex-7_Fuc-1_NeuAc-2
HexNAc-6_Hex-7_Fuc-2
HexNAc-6_Hex-7_Fuc-3
HexNAc-6_Hex-7_Fuc-4
HexNAc-6_Hex-7_NeuAc-2
HexNAc-6_Hex-7_NeuAc-3
HexNAc-6_Hex-7_NeuAc-4
HexNAc-6_Hex-9
HexNAc-7_Hex-3
HexNAc-7_Hex-3_Fuc-1
HexNAc-7_Hex-4_Fuc-1
HexNAc-7_Hex-6
HexNAc-7_Hex-6_NeuGc-2
HexNAc-7_Hex-7
HexNAc-7_Hex-7_Fuc-1
HexNAc-7_Hex-7_Fuc-1_NeuAc-3
HexNAc-7_Hex-7_NeuAc-3
HexNAc-7_Hex-8
HexNAc-7_Hex-8_Fuc-1
HexNAc-7_Hex-8_Fuc-1_NeuAc-4
HexNAc-7_Hex-8_Fuc-3
HexNAc-7_Hex-8_NeuAc-1
HexNAc-7_Hex-8_NeuAc-2
HexNAc-7_Hex-9
HexNAc-8_Hex-11
HexNAc-8_Hex-3
HexNAc-8_Hex-3_Fuc-1
HexNAc-8_Hex-4
HexNAc-8_Hex-5
HexNAc-8_Hex-5_Fuc-1
HexNAc-8_Hex-7
HexNAc-8_Hex-8
HexNAc-8_Hex-9
HexNAc-8_Hex-9_Fuc-1
HexNAc-9_Hex-3
HexNAc-9_Hex-4_Fuc-1
HexNAc-9_Hex-6
HexNAc-9_Hex-6_Fuc-1

Supplementary Figure S2. Distribution of glycan assignments in synaptosome samples, related to Figure 4. All false discovery rate (FDR)-filtered unique glycans assigned using MSFragger for synaptosome samples are shown. Annotated glycans are shown in colors corresponding to those used in the main text, while the non-annotated “Excluded” category contains all compositions listed in the lower panel. While included glycans make up a minority of all unique detected *N*-glycan compositions, they represent the vast majority (86%) of all detected glycoPSMs. Glycan annotations were made according to published accounts of the mouse brain *N*-glycome (Williams et al., 2022), which demonstrate a predominance of mannosylated and fucosylated complex glycans along with a paucity of *N*-linked sialylated glycans and a predominance of *O*-linked sialylated glycans. A small minority of glycans that correspond to fucosylated glucosyl-mannosyl and high-mannose glycans were annotated but not included in the “Fuc1” category, which was reserved for fucosylated complex glycans. Many non-annotated glycan IDs do not correspond to previously observed compositions of *N*-linked glycans, suggesting that they may represent tryptic glycopeptides modified with both *N*- and *O*-linked glycans and thus cannot confidently be assigned to the *N*-glycoproteome.

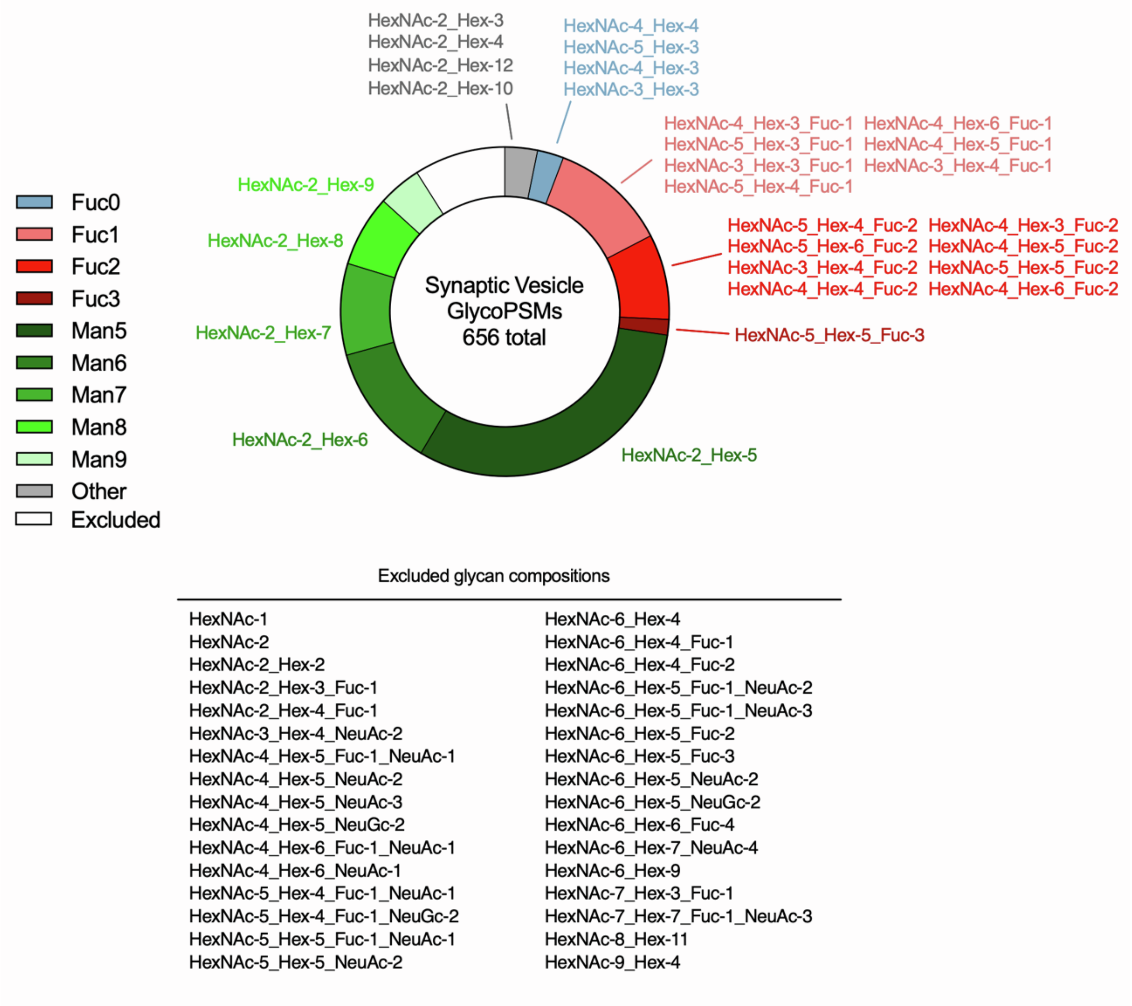

Supplementary Figure S3. Distribution of glycan assignments in SV samples, related to Figure 4. All FDR-filtered unique glycans assigned using MSFragger for SV samples are shown. Included glycans are shown in colors corresponding to those used in the main text, while the "Excluded" category contains all compositions listed in the lower panel. While included glycans make up a minority of all detected *N*-glycan compositions, they represent the vast majority (90%) of all detected SV glycoPSMs. Glycan annotations were made according to published accounts of the mouse brain *N*-glycome (e.g., Williams et al., 2022), which demonstrate a predominance of mannosylated and fucosylated complex glycans along with a paucity of *N*-linked sialylated glycans and a predominance of *O*-linked sialylated glycans. Many non-annotated glycan IDs do not correspond to previously observed compositions of *N*-linked glycans, suggesting that they may represent tryptic glycopeptides modified with both *N*- and *O*-linked glycans and thus cannot confidently be assigned to the *N*-glycome.

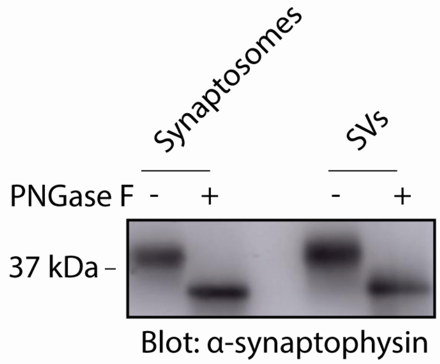

Supplementary Figure S4. Immunoblot confirmation of deglycosylation by PNGase F, related to Figure 5. Synaptosome and SV samples were subject to deglycosylation with PNGase F according to conditions used for glycan release and labeling (see main Fig. 5) and subjected to SDS-PAGE followed by immunoblot with anti-synaptophysin antibody. Deglycosylation of this synaptic vesicle glycoprotein causes an increase in SDS-PAGE mobility. Following treatment with PNGase F, all synaptophysin has been converted to the higher-mobility species, indicating quantitative deglycosylation for both SVs and synaptososomes under these conditions.

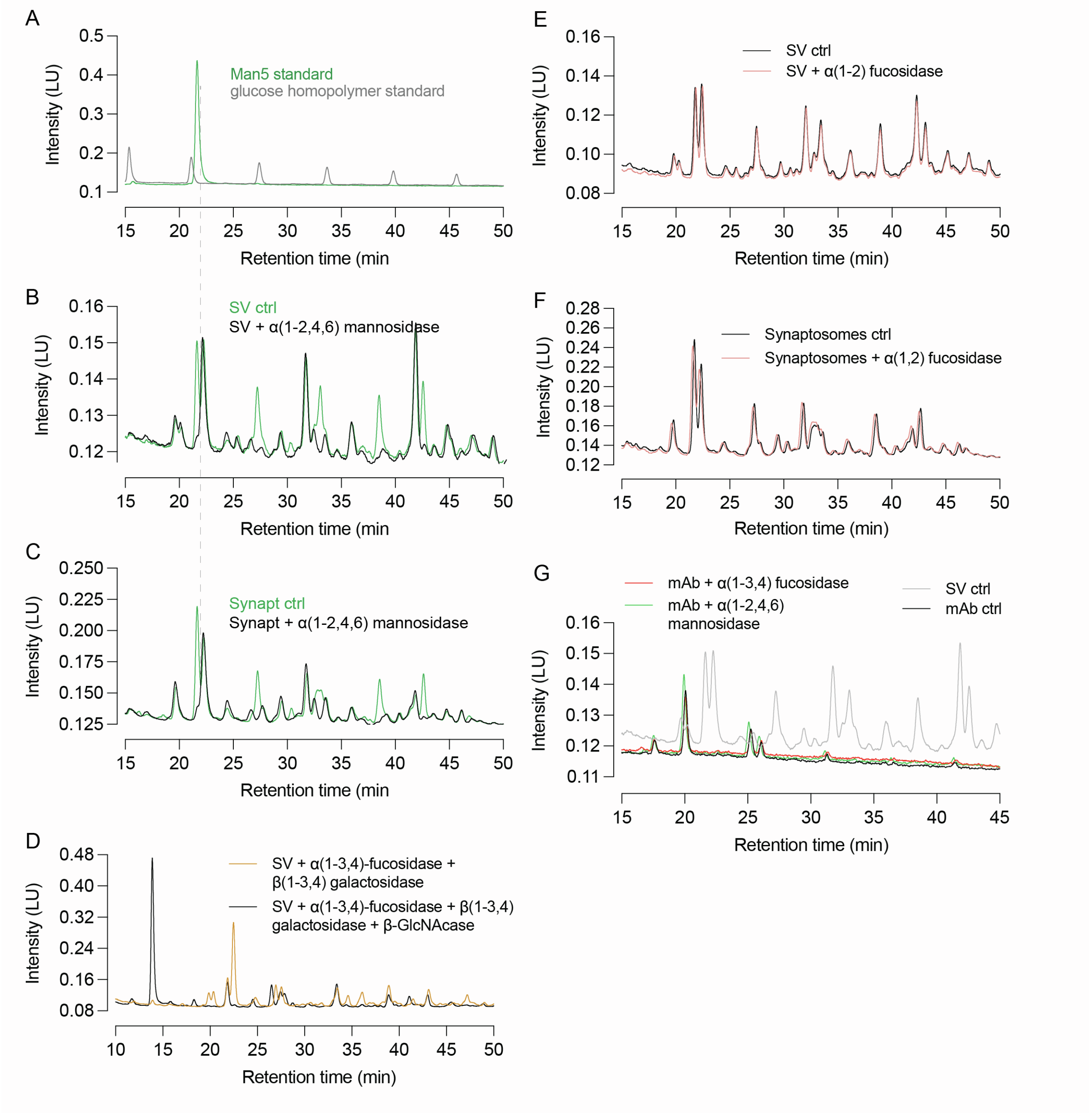

Supplementary Figure S5. Supporting information for HILIC-FL studies, related to Figure 5. (A) A Man5 standard and a linear glucose homopolymer standard were labeled with procainamide and analyzed by HILIC HPLC. Individual peaks in grey correspond to glucose polymers of increasing length, with each additional glucose in the pictured peaks causing an increase in retention time by 6.1 ± 0.2 (mean ± SD) minutes. The authentic Man5 standard labeled with procainamide is shown in green. (B) HILIC-HPLC trace of SV *N*-glycan sample labeled with procainamide with or without treatment with α(1-2,4,6) mannosidase. Mannosidase-sensitive peaks occur at the same position as the Man5 standard and at evenly spaced increasing retention times of ~0.7-1 glucose units, consistent with well-established *N*-glycan structures containing up to 9 mannose residues in a branching configuration. (C) as in (B) but with synaptosome glycans. (D) The peak resulting from galactosidase treatment of φ* (see Figure 5) is further sensitive to cleavage by β-GlcNAcase, confirming that φ is an elaboration of a GlcNAc-bearing complex *N*-glycan. (E) α(1-2) fucosidase does not cause shifts in elution time of synaptosome glycans, consistent with a minimal contribution of fucose α(1-2) galactose sugars to synaptosome glycans and the absence of the enzymes (Fut1, Fut2) that catalyze the addition of fucose at the 2-position of galactose. (F) as in (E) but for SV *N*-glycans. (G) SV2 mAb *N*-glycans are insensitive to α(1-3,4) fucosidase and α(1-2,4,6) mannosidase and elute at times distinct from major SV *N*-glycans.

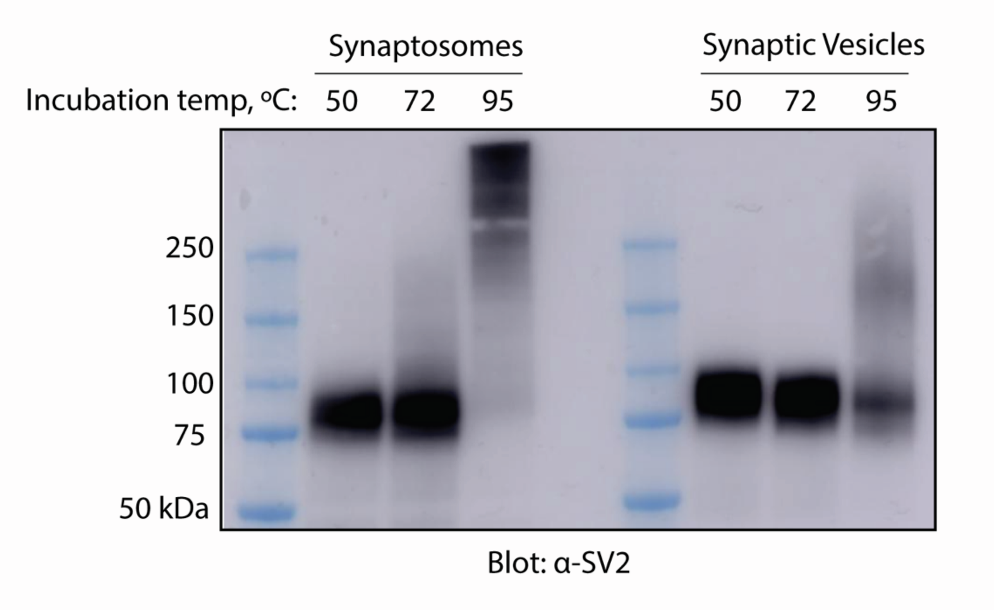

Supplementary Figure S6: The apparent molecular weight of SV2 increases with application of heat, related to Figure 6. Synaptosome or SV samples were combined with SDS sample buffer containing DTT and heated for 15 minutes prior to SDS-PAGE and immunoblot with anti-SV2 antibody. SV2 immunoreactivity shifted to higher molecular weight in a graded fashion with the application of increased heat.
